# Genetic and Molecular Regulation of Seed Size in *Brassica napus*: Current Knowledge and Breeding Perspectives

**DOI:** 10.3390/ijms27219465

**Published:** 2026-10-23

**Authors:** Jin Wang, Sana Ashraf, Yu Han, Xinyao Long, Wenlong Jiang, Hasan Huseyin Atar, Mushtaq Ahmad, Li Ma, Qinxue Zhang

**Affiliations:** 1 School of Biological Science and Technology, Jiangsu University, Zhenjiang 212013, China; 2 Department of Botany, University of Agriculture Faisalabad, Faisalabad 38000, Pakistan; 3 Faculty of Agriculture, Ankara University, Ankara 06110, Türkiye; 4 Biodiversity and Bioenergy Research Centre, Department of Plant Sciences, Quaid-i-Azam University Islamabad, Islamabad 45320, Pakistan; 5 State Key Laboratory of Aridland Crop Science, Gansu Agricultural University, Lanzhou 730070, China

**Keywords:** *Brassica napus*, seed size, research progress, future prospects

## Abstract

*Brassica napus* L. (rapeseed), a member of the family *Brassicaceae*, is the world’s third most important oilseed crop for edible oil production, after soybean and palm. Quantitative trait locus (QTL) mapping is a powerful strategy for dissecting the genetic basis of seed weight. In this review, Previous studies summarized a total of 1905 candidate genes associated with seed weight in *Brassica napus*. Five candidate genomic regions harboring 204 seed-weight-related genes were identified, among which 21 genes were characterized as differentially expressed genes via transcriptomic analysis. Clustered regularly interspaced short palindromic repeats/CRISPR-associated protein 9 (CRISPR/Cas9) mutant libraries targeting genes related to silique and seed size have been constructed in *Brassica napus*, revealing that *BnaHRD* genes play critical roles in seed development. These differentially expressed genes are involved in developmental processes, cell division. and nutrient storage, supporting their functions in seed size regulation. *SHB1* and *HAIKU2* modulate endosperm proliferation through the IKU pathway, thereby affecting seed weight. *BZR1* (brassinazole-resistant 1) acts as a master regulator of seed growth via maternal tissues. Thidiazuron (TDZ), a cytokinin analogue, simultaneously promotes cell proliferation and cell expansion, thereby increasing cell number and cell size. Multiple phytohormone signaling pathways, including auxin, gibberellin (GA), and brassinosteroid (BR), as well as transcription factors such as *LEC2*, participate in the regulatory network of seed size. Here, we review current advances in the genetic and molecular regulation of seed size and discuss future perspectives for rapeseed breeding.

## 1. Introduction

Rapeseed (*Brassica napus* L.) belongs to the *Brassicaceae* family. This plant is commonly referred to as “oilseed rape”. Biomass allocation is a key component of the functional-structural feedback mechanism. To improve the simulation accuracy of the aboveground biomass allocation coefficient model for the main stem, primary branches, and their respective stems, leaves, and pods, this study quantifies the relationship between the biomass allocation coefficients of major aboveground organs and the days of physiological development (DPD) [1]. Rapeseed seeds have a wide range of uses in many countries. *Brassica napus* belongs to the *Brassicaceae* family, which is one of the largest plant families in the world with about 435 genera and 3675 species [2]. Rapeseed (*Brassica napus*), also known as “rape”, “rapa”, “oilseed rape”, is a flowering plant characterized by bright yellow flowers. Its name is derived from the Latin root word rapa or rapum, meaning turnip. In ancient literature, turnips and rapeseed were distinguished by the adjective’s “round” and “long (root-like)”, respectively. In 2021, *Brassica napus* accounted for approximately about 12.1% of global vegetable oil production and ranked among the world’s leading oilseed crop [3]. Rapeseed oil is one of the oldest vegetable oils; however, its consumption was historically limited because of the high concentration of erucic acid in the oil and glucosinolates in the meal but its consumption was limited [4,5]. This plant can grow to a height of 50–200 cm and can be incorporated into the soil or used as bedding after harvesting.

Currently, marker-assisted selection (MAS) and transgenic breeding are commonly used to improve rapeseed. Rapeseed was among the first crops to benefit from biotechnology. The rapeseed (genome AACC, 2n = 38) belongs to the *Brassicaceae* family, which contains 3000 other species. It is a product of spontaneous hybridization between *Brassica rapa* (AA, 2n = 20) and *Brassica oleracea* (CC, 2n = 18). Rapeseed has been cultivated in ancient civilizations in Asia and the Mediterranean region, and its oil was once used for lighting. In one study, a genome-wide association analysis (GWAS) was conducted using a germplasm bank containing 302 accessions. The QTL mapping results showed that six QTLs were located on chromosomes A01, A04, A08, and A09 in both experiments. Among them, two major QTLs, qSSC/21GY.A04-1 and qSSC/21NJ.A08-1, explained 12.92% and 10.18% of the phenotypic variation, respectively. Furthermore, a genome-wide association study (GWAS) identified eight single-nucleotide polymorphisms (SNPs), which explained between 5.62% and 10.18% of the phenotypic variation [6]. Rapeseed cultivation dates back to ancient times in India, where it was reportedly practiced as early as 2000 BC. Cultivation in Europe began in the 13th century. Early rapeseed varieties originated in Canada and were used by the Allied Navy as a ship lubricant. After World War II, Western countries began to fully utilize the edible value of rapeseed. At that time, due to oil shortages, various oilseed crops were tested in western Canada, including sunflowers, safflower, soybeans, and annual rapeseed. Silencing the *FAD2* gene using RNA interference significantly increased the oleic acid content in rapeseed. The high-oleic acid transgenic seeds maintained normal germination and growth characteristics [7]. Annual rapeseed adapted to large areas of western Canada and showed high yields.

As one of the main sources of edible vegetable oil [8], increasing rapeseed yield is crucial for ensuring oil supply. Seed weight is a key factor determining rapeseed yield, and it is significantly positively correlated with seed size. Besides providing shelter for seed development, the siliques of rapeseed also play a crucial role in the accumulation of storage substances during the later stages of seed development [9,10]. Variations in silique traits (such as length and width) directly or indirectly affect yield [11]. Therefore, silique traits, like seed weight, have long been considered important targets for rapeseed genetic improvement. Seed size/weight is one of the key traits associated with plant adaptability and crop yield. *Brassica napus* seed size exhibits extensive natural variation even among different germplasms of the same species, which is of great value for evolutionary research and crop improvement. However, the regulatory mechanisms of this natural variation remain unclear.

Oils and fats are one of the three major nutrients essential for human survival. Rapeseed oil accounts for approximately 15% of the world’s total production [12]. High-quality rapeseed oil, particularly of the *Brassica napus* variety, is low in saturated fatty acids and is considered one of the healthiest edible vegetable oils. Rapeseed is mainly produced in Asia, North America, and Europe. According to data from the USDA Foreign Agricultural Service (World Agricultural Production: http://www.fas.usda.gov/data/world-agricultural-production) (accessed on 10 November 2025), in 2015, these three regions accounted for 31%, 24%, and 32% of the world’s total production, respectively. China and India are the two largest rapeseed producers in Asia. China’s annual rapeseed planting area is approximately 7 million hectares, accounting for about 21% of the world’s total production. Rapeseed oil is also China’s largest domestic source of edible vegetable oil (http://www.fas.usda.gov/data/world-agricultural-production, accessed on 10 November 2025). As the world’s third-largest oilseed crop, rapeseed serves as both edible oil and a source of renewable energy [13,14]. Furthermore, due to the high protein content of its oilseed meal, it can also be used as livestock feed [15]. With the increasing demand for vegetable oils and the decreasing area under rapeseed cultivation, increasing rapeseed yield has been a primary goal of rapeseed breeding [16]. Seed weight is one of the three direct components of rapeseed yield (number of siliques per plant, number of seeds per silique, and seed weight) and a key factor in increasing rapeseed yield [17,18]. Oil yield is a primary target trait in rapeseed breeding. Rapeseed oil production efficiency depends on seed oil content and yield [19]. Therefore, these two factors are important breeding objectives for improving rapeseed oil yield. Over 60 years of traditional breeding in China has increased rapeseed yield by 2–3 times compared to the initially introduced varieties with a yield of approximately 750 kg·hm^−2^ [20]. Recent studies suggest that increasing oil content may be a more effective way to increase oil yield per unit area; it is estimated that a 1% increase in oil content is equivalent to a 2–3% increase in seed yield.

With the development of biotechnology, the widespread application of second-generation technologies and (SNP) single nucleotide polymorphism markers has greatly promoted research on rapeseed oil content worldwide. Previous studies bred a novel rapeseed mutant (DS3) carrying dual AHAS mutations (Trp574Leu and Pro197Leu). The combination of these mutations significantly enhanced its resistance to sulfonylurea herbicides through a synergistic effect. Furthermore, a KASP marker targeting the Pro197Leu mutation was developed to facilitate MAS [21]. Especially in China, significant progress has been made in rapeseed oil content research and breeding in recent years. Furthermore, increasing seed weight and the number of seeds per pod are effective methods to improve rapeseed yield, but increasing the number of pods per plant is relatively difficult under China’s high-density planting model [22]. Therefore, elucidating the genetic basis of seed weight can not only deepen understanding of seed development but also help improve the breeding efficiency of rapeseed.

Through fine mapping and association analysis, the first QTL simultaneously affecting seed weight and seed length (VSL)—BnaA9. ARF18—was identified on rapeseed chromosome A09. In another study, using a map-based cloning strategy, a QTL related to Seed number per silique (SPS)—BnaC9. SMG7b—was identified on rapeseed chromosome C09 [23]. Furthermore, two other major QTLs related to seed length (VSL) were identified on rapeseed chromosomes C07 (BnaC7.ROT3) and A09 (BnaA9. CYP78A9), respectively, and were cloned and identified using map-based cloning methods [24,25]. Although some candidate genes for silique traits have been cloned and functionally characterized, key candidate genes for silique traits, especially key candidate genes for seed density per silique (SDPS), have not yet been discovered. In recent years, multi-locus genome-wide association study (ML-GWAS) methods, including FASTmrEMMA, ISIS EM-BLASSO, mrMLM, FASTmrMLM, pLARmEB, and pKWmEB, have been developed as effective association analysis methods [1,26]. Combinations of multiple ML-GWAS methods have been shown to significantly and effectively control false positive rates [27,28]. Therefore, ML-GWAS has been used to discover new QTNs in various crops. In wheat, based on 172,711 SNPs, five ML-GWAS models successfully identified new QTLs related to yield in 272 local Chinese wheat varieties [29]. ML-GWAS analysis of 144 maize inbred lines, using 43,427 SNPs for genotyping, identified a large number of significant QTNs associated with embryogenic callus regeneration capacity and 40 candidate genes [30]. In soybean, five ML-GWAS association study methods identified 129 significant QTNs associated with protein content and predicted eight candidate genes involved in protein synthesis and metabolism [31]. Using ML-GWAS, 74 significant QTN hotspots associated with five yield-related traits in rice have been identified [30]. However, no ML-GWAS has been conducted on rapeseed yield-related traits, especially those based on whole-genome resequencing data. Given the efficiency and variety of models available for ML-GWAS, it is expected to identify more novel loci and candidate genes related to yield traits. To identify novel loci for rapeseed yield and related traits, which are complex quantitative traits governed by multiple genes and influenced by environmental conditions. The researchers used six ML-GWAS methods to identify new QTNs based on high-quality SNP data of eight yield traits from 403 rapeseed germplasm resources under three environments. Rapeseed yield components and related traits are complex quantitative traits controlled by multiple genes. In previous studies, several loci or genes related to rapeseed yield traits were identified using QTL mapping and map-based cloning methods [23,25,32,33,34,35]. However, findings from other crop species should not be considered direct evidence of gene function in *Brassica napus*. The discussed genes are classified according to the available evidence, distinguishing functional validation in *B. napus* from association or QTL evidence, expression data, and orthology-based inference.

## 2. Genetic Control of Seed Size

Seed size is controlled by multiple genetic factors. Transcriptome-wide association study (TWAS) data from different developmental stages of the silique [36] were analyzed and [37] compiled into a comprehensive list containing 1905 candidate genes. Ref. [37] targeted 84 genes in 151 gene-edited plants. These genes included 71 genes screened from TWAS data, 11 genes from existing literature, and 2 genes specifically expressed in *Brassica napus* siliques. These edited genes share homology with genes such as *AT2G36450*, *AT5G57790*, *AT5G47330*, *AT3G52115*, *AT5G52050*, *AT1G24150*, *AT5G52600*, and *AT2G43120*. These genes play important regulatory roles in plant growth and development. Notably, the ectopic overexpression of certain genes involved in network development has been shown to improve plant resistance to drought and salinity stress [38]. To complement this approach, a database comprising 395 functionally relevant genes was compiled from the literature on model plants such as *Arabidopsis thaliana* and rice, with a focus on seed size, floral organ size, and total stem width. Furthermore, through transcriptome analysis of Zhongshuang 11 (ZS11), 89 silique wall-specific expression genes were isolated, corresponding to 51 orthologous genes. Seed size, seed weight, and yield should not be confused. Thousand seed weight (TSW), number of seeds per pod, pod length, seed yield, and oil production are interrelated but independent traits. Seed size reflects the geometric dimension of seeds, while thousand-seed weight characterizes grain quality. Although the two are highly correlated, their concepts are different. The final seed yield is the result of the synergistic effect of multiple yield components. In *Brassica napus*, the increase in seed size often has a trade-off effect on traits: an increase in individual grain size is often accompanied by a decrease in the number of seeds per pod or pods per plant, resulting in a yield compensation effect, and ultimately the total yield may not necessarily increase. Therefore, the review needs to distinguish between two types of genes: one that directly regulates embryo and endosperm development to change the size of the seed itself; and another type that indirectly affects yield by regulating flowering, pod development, plant type, and other pathways. The phenotypic effects of various genes and QTLs are environment-dependent, and many loci are only significant in specific environments. Therefore, their multi-environment stability must be evaluated in breeding applications.

**Figure 1:**
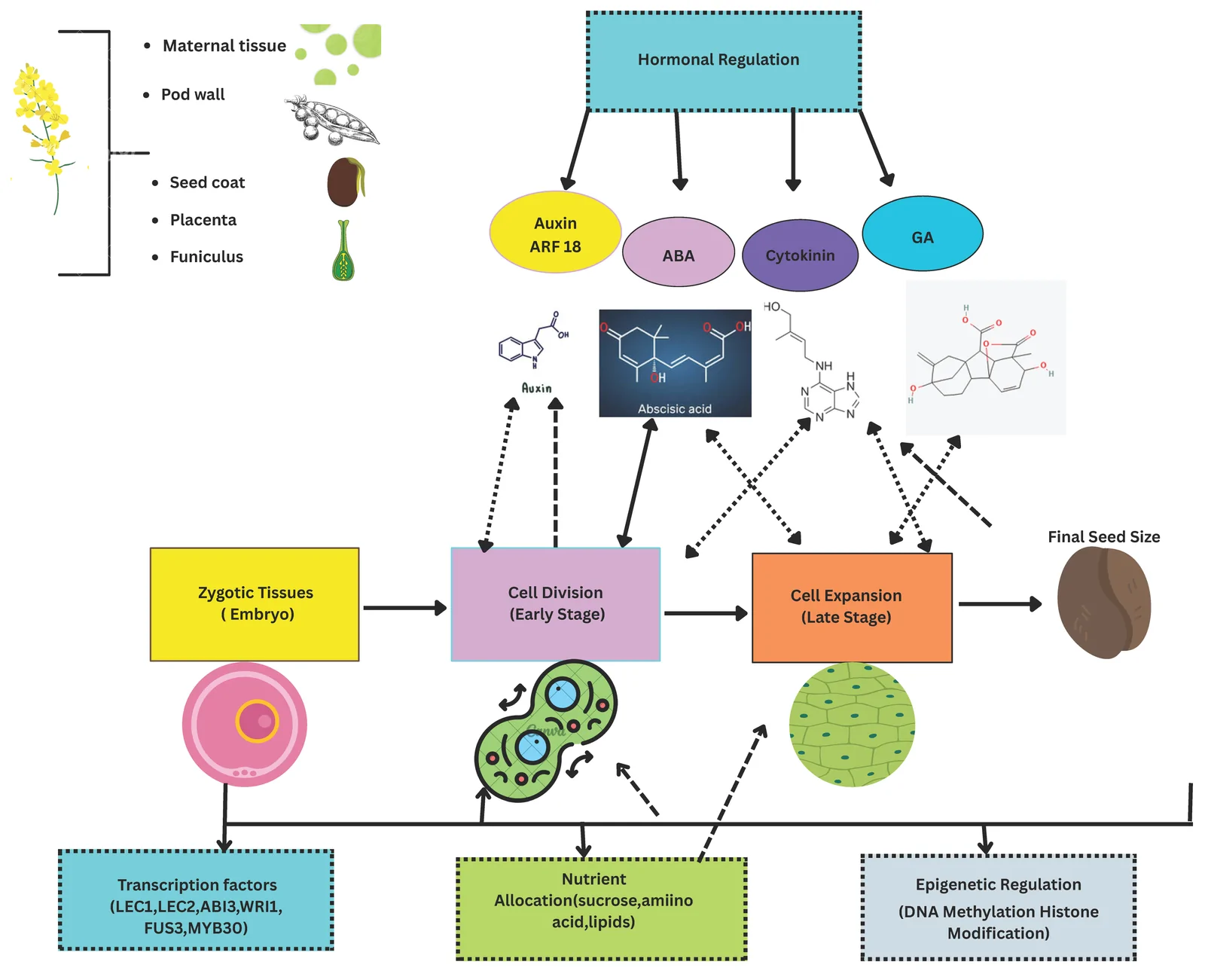
Genetic control of seed size. This diagram illustrates the genetic and hormonal regulatory network controlling seed size at different stages of seed development. Major plant hormones, including auxin (AUX), abscisic acid (ABA), brassinosteroids (BRs), cytokinins (CKs), and gibberellins (GA), regulate cell division, cell enlargement, storage accumulation, and seed maturation through specific signaling pathways and transcription factors. Maternal tissues, embryonic tissues, epigenetic modifications, and key regulatory genes collectively influence seed growth and development. The interactions between hormonal signals, transcription factors, and developmental regulators ultimately determine the final seed size traits, including seed length, width, thickness, and TSW.

Using the pRGEB32-BnU6C01 vector backbone, a CRISPR/Cas mutant library targeting candidate genes in *Brassica napus* related to silique and seed size was constructed. This suggests that *BnaHRDs* may be involved in regulating silique and seed development. Seed growth is synergistically regulated by maternal and zygotic tissues [39,40]. Notably, abscisic acid (ABA) and auxin are interconnected via the ARF2-ANT-COR15A signaling cascade, jointly regulating seed weight [41]. ABA [42], brassinosteroids [43], and cytokinins [44] are located upstream and downstream of the *HAIKU* signaling pathway, respectively, influencing seed size through zygotic regulation. As shown in Figure 1, the genetic and hormone regulatory networks that regulate seed size at different stages of seed development are demonstrated. Pod length and TWS showed similar trends, and the pod length of large-seed recombinant inbred lines (RILs) was significantly greater than that of small-seed recombinant inbred lines (*p* = 1.22 × 10^−12^, 38.2% higher). Considering the trade-off between seed weight and seed number [45], the number of seeds per pod and seed yield per pod. The calculated effects of the maternal parent on seed weight were 0.97 and 0.88 for L_RIL0974 and S_RIL1148, respectively, similar to the values estimated using other rapeseed lines [23].

Seed filling in *Brassica* species depends on photosynthetic products provided by maternal plant tissues, including leaves, green stems, and pod/fruit walls. These photosynthetic products are the major contributors to final seed weight in rapeseed, consistent with previous findings [46,47]. To seek molecular evidence for a causal relationship between pod length and seed weight, QTL mapping and comparisons using the *BnaZNRIL* population [48] were performed. The broad-sense heritability of seed weight and pod length were 0.85 and 0.93, respectively, which were relatively high and consistent with previous findings [49]. From a genetic perspective, QTL co-location is thought to be due to tight linkage or pleiotropic effects [50]. In *Brassica napus*, the auxin-responsive factor *BnaA9.ARF18* negatively regulates seed weight by influencing silique growth in the auxin signaling pathway. Several mutants affecting seed weight have been identified in *Brassica napus*, such as zy72360, R1 [33], G-42, and 7-9 [51] 631 (131 upregulated genes and 500 downregulated genes), and 486 (256 upregulated genes and 230 downregulated genes) differentially expressed genes (DEGs) were identified in the pod wall and seeds, respectively. Solexa sequencing provides a more sensitive assessment of gene expression [52]. Two differentially expressed genes, *BnaA09g55530D* and *BnaA08g23390D*, are orthologs of *Arabidopsis CYP78A9* and *CYP72C1*, respectively, and are positive and negative regulators of pod length [53,54].

Furthermore, differentially expressed genes (DEGs) are involved in development, cell division, and nutrient storage. Among development-related genes, the DEG encoding the FIE protein is upregulated in the large seed pool, and the FIE protein is responsible for seed development [55]. In addition, DEGs encoding microtubule-related proteins that regulate the cell cycle, the cell division control protein cdc2 [56], and AtGRF5 (growth regulator 5) [57] have also been identified. More importantly, a total of 11 DEGs are homologous to known seed weight genes in *Arabidopsis thaliana*, including AEP4, AGL62, CHS, CRA1, CRB, EMS1, ETO1, and CYP78A9. Seed weight and size are complex quantitative traits controlled by both maternal tissues and zygotic tissues, including the embryo and endosperm [24]. Comparative analysis showed that homologs of 32 known fruit/seed size genes [58,59,60,61,62,63,64,65,66,67,68,69,70,71] were located within the confidence intervals of the QTLs for seed weight and pod length.

**Figure 2:**
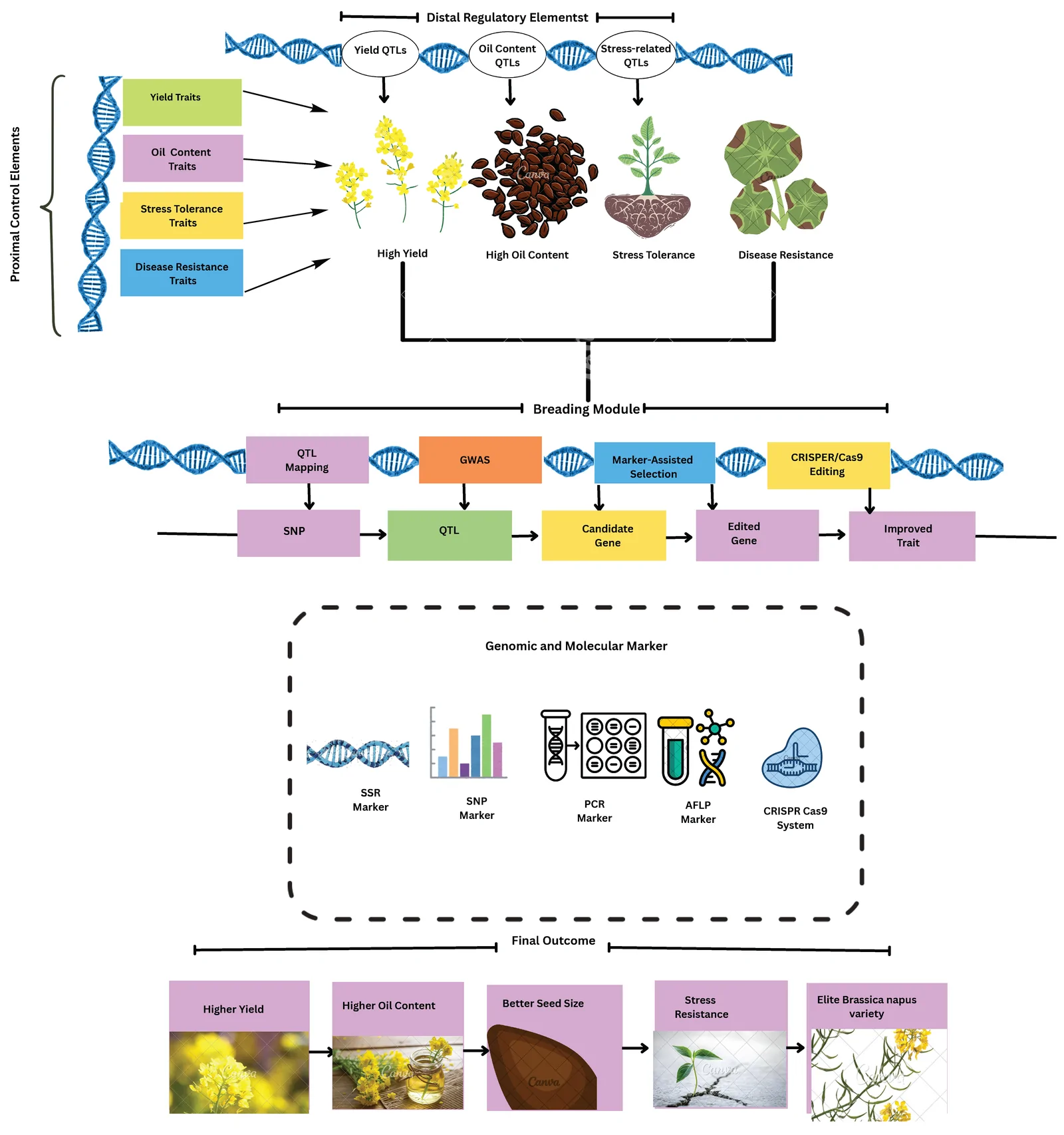
A framework integrating molecular breeding and genome editing technologies to improve traits in rapeseed (*Brassica napus*). The diagram illustrates the relationships between regulatory genomic elements, QTL, molecular markers, and modern breeding techniques aimed at improving agronomic traits. Proximal and distal regulatory elements influence key traits such as yield, oil content, stress resistance, and disease resistance through QTL associated with yield, oil content, and stress.QTL mapping and GWAS help identify significant single nucleotide polymorphisms (SNPs), QTL, and candidate genes, which can then be used for MAS and CRISPR/Cas9-mediated genome editing. Genomic and molecular tools such as SSR, SNP, PCR, AFLP markers, and CRISPR/Cas9 support the discovery and validation of traits. The integration of these methods enables the breeding of improved rapeseed varieties with enhanced yield, oil content, grain size, stress resistance, and overall agronomic traits.

Many QTLs associated with TSW have been located in *Brassica napus* [72,73]; however, the function of only a few genes has been characterized. Four homologous genes of AtUBP15 (AT1G17110) were identified in *Brassica napus*: *BnaUBP15.A06* (*BnaA06G0122700XY*), *BnaUBP15.C05* (*BnaC05G0138100XY*), *BnaUBP15.A08* (*BnaA08G0267100XY*), and *BnaUBP15.C08* (*BnaC08G0236200XY*). Many ubiquitination pathway genes involved in seed size regulation have been identified in *Arabidopsis thaliana*, such as *AtEOD1*, *AtUBP15*, *AtDA1*, and *DA2* [62,74,75,76]; however, the regulatory mechanisms of seed size in *Brassica napus* remain poorly understood. Ref. [72] constructed a BnaEOD3 gene knockout mutant in *Brassica napus* using the CRISPR/Cas9 system. Their results showed that different combinations of mutations in the four homologous copies led to phenotypic changes, including shorter siliques, smaller seeds, and increased SPS levels, with the mutant showing the most significant changes when all four copies were knocked down. Notably, seed size is influenced by the coordinated development of the embryo, endosperm, and seed coat—three key components [31]. Recently, researchers detected 15 QTLs associated with seed weight, among which the major QTL qSW-A03 was identified in a 59 kb genomic region through fine mapping [77]. Although QTLs associated with seed weight have been reported in *Brassica napus*, the functions of only a few genes have been elucidated. To date, only two genes, *BnaA9.CYP78A9* and *BnaA9.ARF18*, have been cloned using QTL-based or map-based cloning methods [25,33]. Furthermore, *SHB1* and *HAIKU2* increase final seed weight by regulating endosperm proliferation in the IKU pathway [78]. A total of 204 genes were annotated within the candidate regions, including 103 non-synonymous mutants and 26 frameshift mutants identified among parental lines. As shown in Figure 2, the interrelationships between regulatory genomic elements, QTLs, molecular markers, and modern breeding techniques are aimed at improving agronomic traits.

By aligning the bulked segregant analysis sequencing (BSA-seq) Bulked-segregant analysis sequencing reads of R140 and ZS11 with the reference genome of *Brassica napus*, we further characterized these genes, identifying three genes with frameshift mutations (*BnaA06G0376100ZS*, *BnaA09G0520900ZS*, and *BnaA09G0521300ZS*), four genes with nonsynonymous mutations (*BnaA06G0371700ZS*, *BnaA06G0374700ZS*, *BnaA06G0378900ZS*, and *BnaA09G0522100ZS*), and one gene exhibiting an upstream mutation (*BnaA09G0559300ZS*). Using a homology-based cloning approach, Previous studies identified several genes associated with seed weight, such as *BnDA1*, *BnaC3.UPL3*, *BnRRF*, *BnEOD1*, *BnaWRKY10*, *BnUBP15*, and *BnaARF2* [79,80,81,82,83]. Previous studies screened five candidate genomic regions containing 204 seeds related to seed weight and integrated transcriptome data to ultimately identify 21 differentially expressed genes within these regions.

In summary, this suggests that R140 and R1 may be different variants of the same gene associated with seed weight in *Brassica napus*. QTL analysis has proven to be a powerful tool for elucidating the genetic mechanisms regulating seed weight in *Brassica napus* [14,17,18,32,84,85,86,87,88,89,90]. Using the basic local alignment search (BLAST) version 2.15.0 tool, 1643 SNP markers were aligned to pseudochromosomes of *Brassica napus* to identify the most significant alignments. Of the 29 QTLs, 24 were integrated through meta-analysis, forming 6 reproducible shared QTLs, named cqSW.A03-1, cqSW.A03-2, cqSW.A07-1, cqSW.A07-2, cqSW.A08-1, and cqSW.C02-1. Candidate gene identification for *cqSW.A03-2*: Within a 61.6 kb region, based on annotations from the “Darmor-bzh” reference genome, 18 predicted coding sequences (CDS) were identified. The significantly associated single nucleotide polymorphism (SNP) SA03_18863834 was located within the region encoding the histidine kinase gene (*BnaA03g37960D*). *BnaA03g37960D* encodes a histidine kinase homologous to *Arabidopsis thaliana* histidine kinase 2 (AHK2), which has been shown to be involved in the regulation of *Arabidopsis* seed size [68]. *BnaA03g37960D* has relatively high expression levels in the ovary and early stages of seed development.

*BnaA03g37960D* is a potential candidate gene at the cqSW.A03-2 locus. Two closely linked QTLs, cqSW.A09-1 and cqSW.A09-2, were identified, which may be identical to the major pleiotropic QTLs for seed weight and silique length previously identified in studies of the A09 linkage group [87,88]. Integrating multiple factors, including seed morphology and cytological analysis, revealed that seed size plays a major role in determining seed weight [91], consistent with the high correlation between seed weight and seed size in this study. Seed size and the grain-filling process determine final seed weight [92]. Cytokinin signaling is involved in the regulation of plant growth and development, stress tolerance, and crop yield [93,94]. Comprehensive display of candidate genes in rapeseed QTL, molecular mechanisms, and their impact on seed traits (Table 1).

TDZ is a highly efficient synthetic phenylurea plant growth regulator that not only possesses auxin-like activity but is also a cytokinin analog [101]. Studies have shown that overexpression of the ANT gene leads to seed enlargement [102]. In particular, auxin biosynthesis and signal transduction in plants largely control seed size [103]. Furthermore, brassinolide resistance gene 1 (*BZR1*) is believed to play an important role in seed growth regulation through maternal tissues [104]. Some studies have shown that specific seed development pathways regulated by brassinolide (BR) transcription lead to changes in seed size and shape [43]. Research has found that TDZ has a significant effect on seed size; seeds from different parts of the plant treated with different concentrations of TDZ were significantly larger than those in the control group. TDZ treatment increased cell size and number, which may be one reason for seed enlargement. The *AP2*, *ARF2*, *BnaA9.ARF18*, and *AGB1* genes play a negative regulatory role in seed size regulation, while the *AGG3*, *ANT*, *GS2*, and *RGA1* genes play a positive regulatory role. *TDZ* may affect seed growth and regulate seed size by promoting or inhibiting the transcription of genes related to seed size. As shown in Table 2, detailed information on hormone regulation during the development of rapeseed is presented.

Seed size is an important agronomic trait that affects not only a plant’s adaptability to environmental stress but also crop quality and yield [105,106]. The transparent seed coat (*TT*) gene is also considered an important candidate gene explaining the differences in seed color in *Brassica* species [107,108,109,110,111]. Recent studies have found that KIX8/9 and PPD1/2 promote seed enlargement by facilitating cell proliferation and elongation, indicating that the KIX-PPD-MYC-GIF1 pathway plays a role in controlling seed size [112]. ABI5 can regulate seed dormancy, germination, and seedling growth [113]. The AGG3 loss-of-function mutant produces smaller seeds and a reduced number of seeds per pod [114,115,116]. Previous studies have shown that *EOD3* plays a crucial role in controlling seed size and silique length in various crops [117]. Compared to the wild type, the mutant exhibits significantly increased seed number and seed weight per plant, which is thought to potentially improve rapeseed yield.

**Figure 3:**
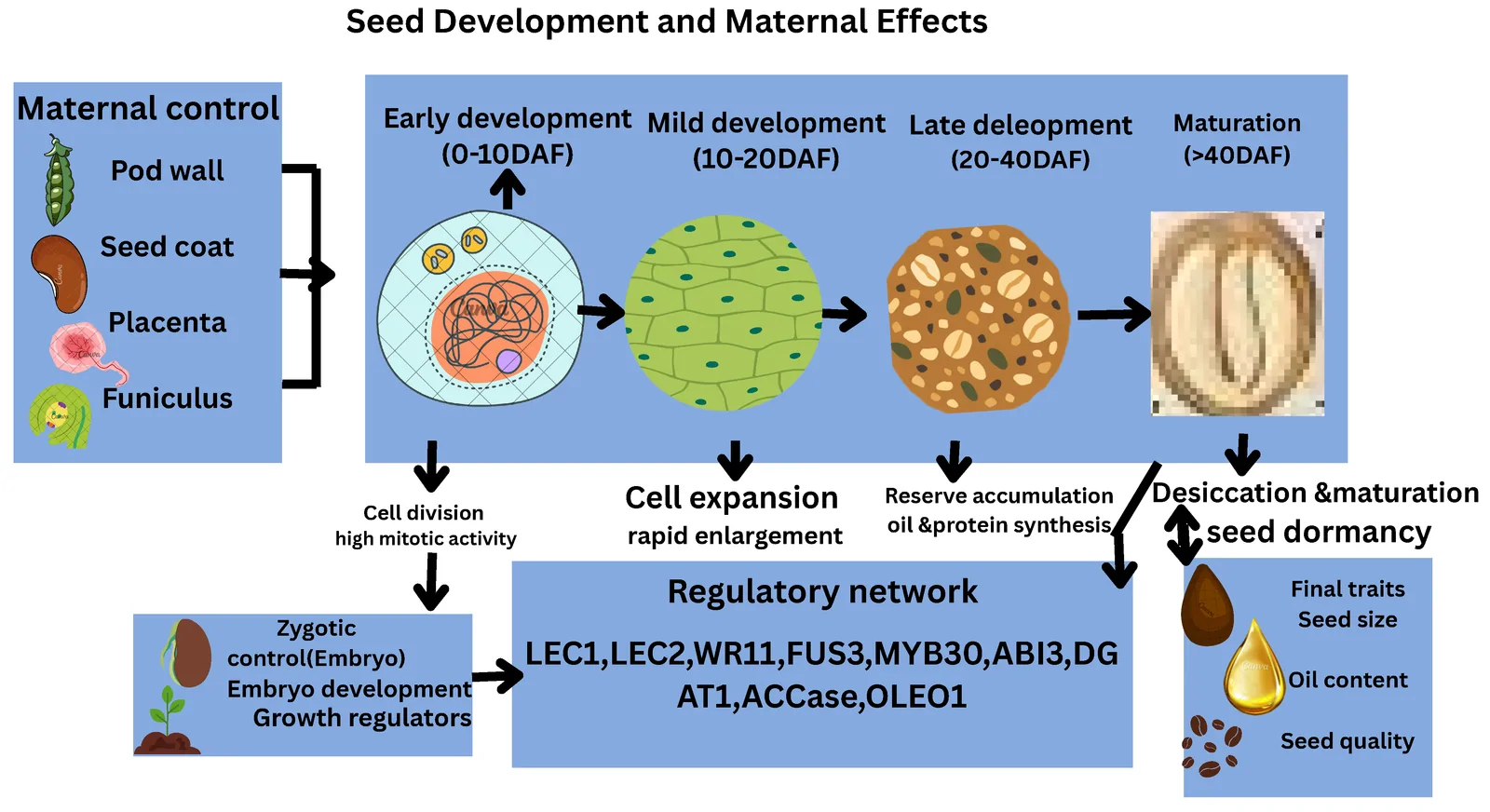
The influence of seed development and maternal effects on seed size, composition, and quality. This schematic illustrates the process of seed development from early stages to maturity, highlighting the coordinated regulation of the process by maternal tissues (via the pod wall, seed coat, placenta, and funiculus) and the zygote (i.e., the embryo). The developmental progression encompasses stages such as cell division, cell elongation, storage reserve accumulation, desiccation, and maturation; these stages are governed by growth regulators and molecular networks, ultimately determining seed size, oil content, and quality.

The role of *BnaA04.EOD3* in seed development is likely relatively small. The newly developed CRISPR/Cas9 technology provides a powerful approach to effectively overcome this limitation and promotes progress in both basic and applied research on rapeseed. To date, this technology has been successfully used to improve several important agronomic traits [118,119,120,121,122]. These results strongly demonstrate that members of the *BnaEOD3* gene in *Brassica napus* may play a differential role in seed development. In this study, seed coat cell size, seed coat cell number, cotyledon cell size, and cotyledon cell number in these lines were all significantly/highly correlated with seed weight (*p* < 0.0001/r = 0.6912, *p* < 0.0001/r = 0.8600, *p* < 0.0001/r = 0.589, and *p* < 0.0001/r = 0.7894). Seed weight was also highly significantly correlated with seed size (including seed diameter, seed surface area, and seed volume) and showed synergistic variation. The number of cells in the seed coat and hypocotyl was the main factor influencing seed length variation. Large-seeded varieties have an average of 10% more seed coat cells and 18% more hypocotyl cells than small-seeded varieties, while the average length of these cells is only 6% and 4% longer, respectively. However, only a few cloned genes, such as *CYP78*A5 [123], *ARF2* [124], *TTG2* [125], *AP2* [126,127], and *EOD3/CYP78A6* [117], regulate seed size/weight in *Arabidopsis* through maternal pathways, while most cloned genes regulate seed size/weight through non-maternal pathways [128]. The influence of seed development and maternal effects on seed size, composition, and quality (Figure 3). Genomic and molecular tools such as SSR, SNP, PCR, AFLP markers, and CRISPR/Cas9 support the discovery and validation of traits. The integration of these methods enables the breeding of improved rapeseed varieties with enhanced yield, oil content, grain size, stress resistance, and overall agronomic traits. As shown in Figure 2, an integrated framework aims to improve the agronomic traits of *Brassica napus* through molecular breeding and genome editing techniques.

**Table 2:** Hormonal regulation in the development of rapeseed.

Hormone/Pathway	Major Gene Involved	Species of Original Evidence	Evidence Type	Role in Seed Development	Phenotypic Effect	Regulatory Interaction	Main References
Auxin Signaling	*ARF2*, *ARF18*, *ANT*	*B. napus* (ARF2, ARF18); *A. thaliana* (ANT)	1, 2, 4	Controls cell division and cell elongation during seed and silique development	Perturbation of these genes increases seed size; ARF2 and ARF18 act as negative regulators	Interacts with the ABA signaling pathway	[33]
ABA Signaling	*ABI3*, *ABI5*, *BnABA3*	*B. napus* (BnABA3); *A. thaliana* (ABI3, ABI5)	3, 4	Mediates seed maturation, desiccation tolerance, and seed dormancy	Heat stress reduces seed storage reserve accumulation	Cross-talk with the ARF2–ANT auxin regulatory module	[129]
Brassinosteroids	*BZR1*, *ROT3*	*B. napus* (ROT3); *A. thaliana* (BZR1)	2, 4	Maternal tissue-dependent regulation of silique and seed growth	Promotes cell expansion, leading to enlarged seeds and siliques	Coordinates cell expansion in seed coat and silique wall	[24]
Cytokinin	*AHK2*, *CKX5*	*A. thaliana*	4	Modulates cell proliferation during early seed development	Elevated cytokinin signaling increases seed weight	Functionally linked to the HAIKU endosperm pathway	[130]
Gibberellin (GA)	*RGA1*	*A. thaliana*	4	Regulates seed growth and cell expansion	Promotes larger seed morphology	Antagonistic regulatory effects against ABA signaling	[131]
HAIKU Pathway	*SHB1*, *IKU2*	*A. thaliana*	4	Governs endosperm proliferation in early seed development	Enhanced endosperm proliferation increases final seed weight	Zygotic regulatory module controlling endosperm growth	[132]
Ubiquitination Pathway	*DA1*, *UBP15*, *EOD1*	*B. napus* (DA1, UBP15); *A. thaliana* (EOD1)	1, 3, 4	Controls organ size via targeted protein degradation	Restricts excessive seed enlargement	Protein turnover signaling to limit cell proliferation	[79]

Note: Evidence Type (same as Table 1).

## 3. QTL Mapping, GWAS, and Genome Mapping

Due to differences in research methods and materials, the influence of genes on seed oil content varies. Previous studies showed that the oil content of *Brassica napus* seeds is influenced by the maternal genotype, while [133] found that the oil content of hybrids is influenced by both embryo type and maternal genotype. Another study by [134] indicated that oil content is jointly determined by embryo type, cytoplasmic and maternal genotype effects, as well as the interaction between genotype and environment. These results suggest that studies conducted under different genetic backgrounds may be able to detect one effect, but the detection of other effects may be limited.

Diversity arrays technology (DArT) markers are generally distributed throughout the genome, although some chromosomes show low or no polymorphism. Approximately 80% (2457/3072) of these markers have been physically mapped to the sequenced genomes of *Brassica rapa* and *Brassica oleracea*. All genetically mapped markers, including DArT markers, and their association with agriculturally significant traits in the mapped populations were investigated. A total of 139 marker regions were identified, which were associated with resistance to Sclerotinia sclerotiorum and Blackleg (causing Blackleg disease), as well as yield-related traits (seed number, seed weight, seed yield, oil yield, number of branches, number of pods, and oil quality). To date, QTL mapping studies conducted by different researchers have shown that 17 of the 19 chromosomes in *Brassica rapa* carry gene loci associated with the control of seed oil content [97].

Traits (glucosinolate content, fatty acid content, and vitamin E), biomass yield, phenological traits (plant height, flowering time, vernalization requirement, maturity time), male sterility, boron use efficiency, and the uptake of nutrients (including boron, calcium, iron, magnesium, copper, zinc, and phosphorus) were analyzed [135,136,137,138]. The marker interval XbrPb-X657955-dFAD2a explained the highest variation (75.6%), while the marker interval brPb-662948-brPb-660893 explained the lowest portions of the variation in linolenic acid and palmitic acid content (13.9%), respectively. Due to the lack of shared markers between the consensus map and the original mapping population, some previously published QTLs could not be precisely identified. Previous studies constructed the first consensus genetic map of the rapeseed genome based on DArT markers. This consensus map contains 1359 markers, covering all 19 chromosomes of *Brassica napus* with an average marker density of one marker per 1.46 cM. It is known that these genome-derived markers introduce a certain degree of redundancy [139]. The CNG methylation-sensitive restriction endonuclease PstI was used to prepare the genome, which produces low-copy-number and single-copy DNA fragments in plants.

This consensus map is applicable to a variety of applications, including the detection of quantitative traits in rapeseed improvement projects. Compared with DArT markers, SSR markers exhibit extensive repetitive sequences and multiple bands (alleles). Previous studies have shown that the approximately 1.2 Gb genome of *Brassica napus* [140] is mainly composed of genome sequences from two ancestral species, which exhibit significant collinearity. DArT sequences (available at http://www.diversityarrays.com/) can be used for comparative genomic analysis, identification of QTLs in predictive breeding (as shown in this study), and identification of candidate genes associated with important agricultural traits. QTL mapping using molecular markers is an important attempt to understand complex quantitative traits. The mapping populations used in this study, such as Ag Castle, Maxol, Monty, Skipton, Ag Spectrum, and Surpass 400, are known to be resistant to *L. maculans* at both the seedling stage (variety-specific, qualitative) and the adult stage (variety-specific and/or variety-nonspecific [137,141,142].

The previously published YE2-DH genetic map was used as the basis for marker saturation, which was located in the major (QTL) quantitative trait locus region for seed color and ADF content previously identified by [143]. SSR, SNP, and RAD marker sequences near the detected QTL peaks were located in the A9 chromosome genome sequence using the *Brassica rapa* genome browser (http://www.phytozome.net/ [144] and the BLAST tool (http://brassicadb.cn/#/) [145], accessed in January 2013. The gene-derived marker BnaCCR1HphI in YE2-DH was located 2.1 cM downstream of CN79, outside the 2-LOD QTL confidence interval. ATPase Bra039228 was first identified as a highly promising candidate gene for localization and function, potentially having a significant impact on seed color variation in the *Brassica napus* population studied in this study [146].

However, comparative mapping between the *Brassica* genome and its close relatives using markers developed from *Brassica rapa* sequence data showed that the genetic map of YE2-DH was enriched in the major QTL region for seed color on chromosome A9. The genetic and physical locations of the SSR markers KBrH092O19 and KBrB025M01, flanking the common fiber compound QTL in ExV8-DH and YE2-DH, were completely consistent with the major QTLs for seed color and ADL content previously found in completely unrelated Chinese rapeseed hybrids [147]. Therefore, improving the meal digestibility of rapeseed lines expressing functionally modified or defective CCR1, CAD, and/or AHA10 enzymes is a promising approach to increasing the total seed value. Previous studies identified a total of 177 and 228 candidate genes, respectively, controlling 16 salt stress coefficients (STCs) in *Brassica rapa* under salt stress during seed germination and seedling stages. Ref. [122] reported that 177 candidate genes were identified during the germination stage and 228 candidate genes were identified during the seedling stage. presence of potential candidate genes controlling oil content within QTL regions in the genome [148,149].

Using markers developed from *Brassica napus* sequence data, the genetic map of YE2-DH was enriched in the major QTL region for seed color on chromosome A9. Therefore, improving the meal digestibility of rapeseed lines expressing functionally modified or defective CCR1, CAD, and/or AHA10 enzymes is a promising approach to increasing the total seed value. BnCKX5 and BnERF3 are considered candidate genes for salt stress response, both exhibiting abundant SNP variation in 505 *Brassica napus* germplasm resources. *BnERF3* is also considered a candidate gene for salt stress response, similarly showing abundant SNP variation in 505 *Brassica napus* germplasm resources. Seed germination rate under different stress conditions is an important indicator of seed germination vigor and resistance [150,151]. There is no significant correlation between germination traits and seedling traits. Although many QTLs and candidate genes associated with seedling salt stress have been identified, no candidate genes have been functionally validated under salt stress conditions in *Brassica napus*.

Previous studies showed that they used GWAS to locate 31 QTLs associated with salt stress by performing GWAS analysis on 16 *Brassica napus* seeds to investigate the genetic basis of salt tolerance in *Brassica napus*. Computer models provide new tools for guiding genetic engineering modifications to improve seed oil content. In *Brassica napus*, various techniques have been used for QTL mapping [136,152,153]. Seed oil content in *Brassica napus* is controlled by multiple QTLs, most of which have additive effects. The genome of *B. rapa* has recently been sequenced [154]. Studies have also found that candidate genes exhibit differential expression under salt stress, drought stress, or ABA treatment [155]. Results indicate that BnCKX5 and BnERF3 play positive regulatory roles in the response of *Brassica napus* to salt stress and mannitol stress. Using 10,658 high-quality SNP markers, a total of 497 SNPs associated with yield-related traits in *Brassica napus* were detected [156]. Based on these high-quality SNPs, six ML-GWAS were performed on eight yield traits in 403 *Brassica napus* accessions under three environments to identify novel QTNs. Under different environments, a total of 4796 genes were mined around 75 stable QTNs identified by ML-GWAS.

Plant growth and development involve many candidate genes, such as *BnaA07g30950D* (auxin efflux carrier), which is involved in maintaining the embryonic hormone gradient and also in the development of the aboveground parts and roots. 1807 genes were identified in the QTN of SW, and 942 genes. Previous studies identified three SW-related candidate genes: *BnaA09g39450D* (cytochrome b561), *BnaA09g39790D* (RNA helicase), and *BnaA09g39950D* (lipase). Through searching differentially expressed genes (DEGs) and candidate genes in ML-GWAS results, Previous studies identified 79 reliable candidate genes, of which 50 and 29 regulate SW and SP, respectively. In rapeseed, many complex traits have been resolved using microarray or resequencing data via single-locus-based GLM or MLM [34,81,156,157].

**Figure 4:**
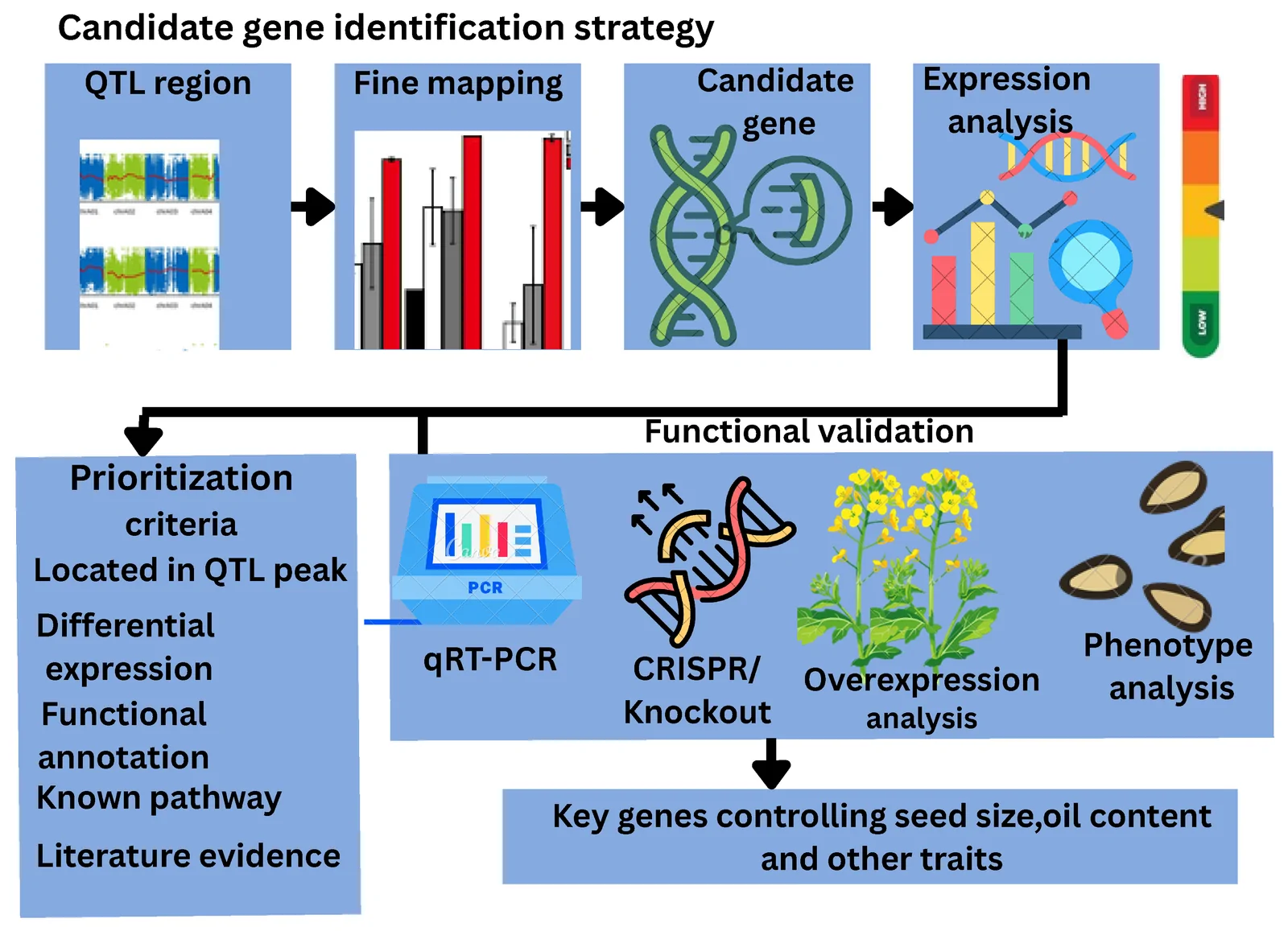
A strategy for identifying candidate genes associated with seed size, oil content, and other agronomic traits. This strategy integrates fine mapping of QTLs and prioritizes candidate genes based on QTL location, differential expression, functional annotation, known pathways, and literature evidence; subsequently, key genes regulating the target traits are identified through expression analysis (qRT-PCR), functional validation (using CRISPR/gene knockout and overexpression), and phenotypic analysis.

In most cases, seed oil content in *Brassica napus* is positively correlated with seed size. Patatin-related phospholipase pPLAIIId has been reported to affect silique size in *Arabidopsis* and *Brassica napus* [158]. Many plant hormones have been reported to be involved in the regulation of seed size, including the auxin pathway, GA signaling pathway, and BR signaling pathway [159]. Differentially expressed genes (DEGs) play important roles in cell division and seed size [160,161]. In recent years, various QTL mapping methods have been used to identify QTLs associated with silique traits in different populations of *Brassica napus* [86,119,162,163,164]. Identifying novel QTLs associated with SDPS and related traits is crucial for fine mapping and cloning, accelerating the breeding of high-yielding rapeseed varieties. To fine-map candidate regions, previous studies developed 383 SSR markers and performed genotyping in two parental lines. From these markers, previous studies screened 15 well-defined and stable polymorphic markers for analysis of 847 F2 individuals to construct a genetic linkage map. As shown in Figure 4, a strategy for identifying candidate genes related to seed size, oil yield, and other agronomic traits.

Previous studies have shown that most agronomic traits of rapeseed, including those related to seeds and siliques, are complex quantitative traits controlled by multiple gene products [7,86,163]. As shown in Table 3, the results of QTL mapping and GWAS analysis in the genetic analysis of rapeseed seed size and yield-related traits are comprehensively presented.

## 4. Yield and Agronomic Traits

As the world’s largest rapeseed producer, China has consistently ranked first in total rapeseed production since the 1980/1981 growing season, except in 2011/2012. Regarding yield and agronomic traits, rapeseed yield in China has increased two to three-fold compared with that of initially introduced varieties, which yielded approximately 750 kg·hm^−2^ [20]. High yield has always been a primary goal in rapeseed breeding. Besides the direct influence of yield components, plant type traits also contribute to rapeseed yield. Selecting and breeding varieties with suitable plant types is an effective strategy for improving the mechanical harvesting rate of rapeseed. Due to significant progress in genetic improvement and breeding, desirable traits such as lodging resistance and high yield have been applied to rapeseed varieties [169,170]. Selecting high-yielding, high-quality varieties suitable for mechanized harvesting is an important way to improve rapeseed production efficiency and planting benefits in the Yangtze River Basin of China [171]. In various planting systems around the world, rapeseed yield shows a significant genotype × environment interaction effect [172,173,174]. High-yielding rapeseed grains mainly result from plant biomass accumulation and a high harvest index [175]. Rapeseed yield is determined by several major yield components, including the number of siliques per plant, the number of seeds per silique, and thousand-seed weight, which are influenced by silique-related traits such as silique length, breadth, thickness, and volume [163]. Pathway analysis of key yield-related traits in *Brassica napus* revealed that the most important yield components included the number of pods per plant, the number of seeds per pod, seed weight, and plant type traits such as plant height and terminal branch angle [120].

**Figure 5:**
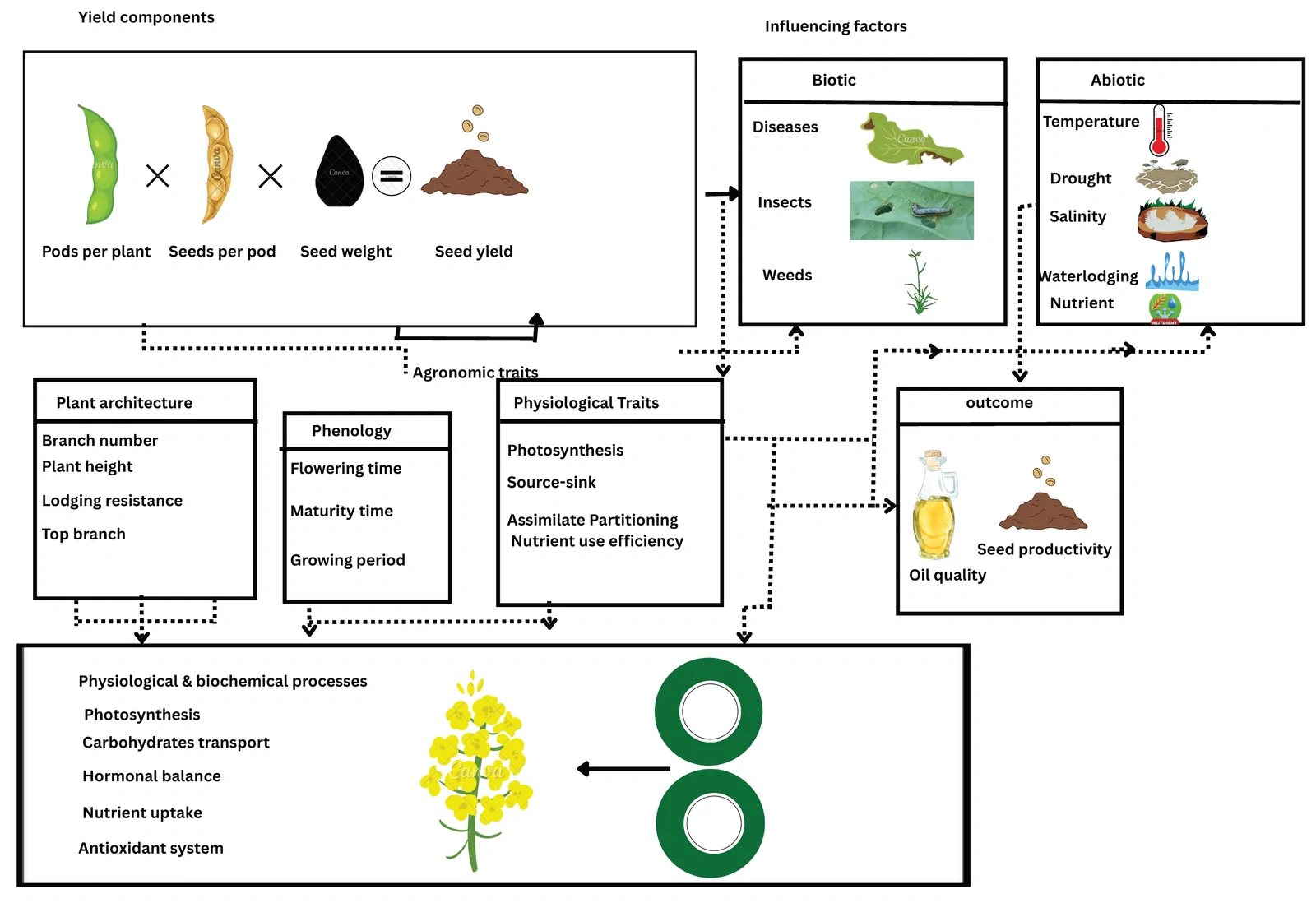
The complex interactions among yield components, agronomic traits, physiological characteristics, and environmental factors that collectively determine seed yield and quality. Yield is influenced by key factors, including the number of pods per plant, the number of seeds per pod, and seed weight, while plant architecture, flowering time, maturity, and nutrient use efficiency affect agronomic traits. Biotic stresses (diseases, pests, and weeds) and abiotic stresses (temperature, drought, salinity, waterlogging, and nutrient limitation) further influence crop productivity by affecting physiological and biochemical processes. The combined effect of these factors ultimately determines oil quality, seed yield, and overall crop performance.

Physiological processes play a crucial role in yield variation and the selection of high-yielding varieties. The relationship between these two factors, known as the harvest index, is the ratio of seed dry matter to aboveground biomass, or economic yield/biomass/harvest index [175]. As reviewed by [176,177], the genetic increase in cereal yield in recent decades has been primarily attributed to the increase in the harvest index. The genotypic correlation between harvest index and yield has been well established [178]. High-yielding rapeseed typically occurs in areas with lower temperatures and abundant, relatively even rainfall. The yield loss caused by mechanized rapeseed harvesting occurs not only during threshing and cleaning, but also when the harvesting time is inappropriate, or when rapeseed collides with the machine during cutting. To minimize nitrogen loss in rapeseed, plant breeders are studying the genetic variation in nitrogen use efficiency [179]. Significant differences exist between rapeseed varieties [180] and genotypes [181]. Generally, the rapeseed life cycle can be divided into seven stages: germination/emergence, leaf growth, stem elongation, bud development, flowering, pod development, and seed development [182]. Average germination time is highly significantly correlated with field performance and seed yield [183]. Flowering is the most critical stage affecting rapeseed yield. Generally, crops are particularly vulnerable to drought stress during flowering, but sensitivity varies among varieties [184]. Under severe drought stress during flowering, due to limited seed bank capacity, up to 65% of the assimilates produced during pod filling are found in the straw. Furthermore, Hansen developed a new recombined double low restorer line for the Ogu-INRA cms in rapeseed (*Brassica napus* L.). It showed that nitrogen fertilizer application has a significant impact on nutrient use efficiency (RUE) of seed growth. Literature review shows that the more component characteristics considered, the more difficult it is to describe yield structure. Furthermore, the reliability of yield analysis depends on the method of collecting or selecting yield traits. Planting density has the greatest impact on seed yield. Seed yield per plant is closely related to the number of pods per plant. Seed weight is less affected by environmental conditions compared to other yield components. Changes in silique traits (such as length and width) will directly or indirectly affect yield [11]. As shown in Figure 5, the complex interactions between yield components, agronomic traits, physiological characteristics, and environmental factors are demonstrated, which collectively determine seed yield and quality.

The use of linkage analysis and GWAS to mine seed size-related loci in *Brassica napus* requires careful interpretation based on population characteristics, experimental environment, and genomic background, and cannot simply equate the localization interval with functional genes. The size of the target population directly determines the resolution and statistical detection effectiveness of QTLs, and small-scale populations often only yield low-resolution QTLs with larger confidence intervals; These early QTLs can only roughly identify candidate regions and cannot be equated with precise mapping or functional validation of causal genes. The environmental stability of loci is another key consideration, as many seed size-related QTLs are regulated by genotype-by-environment interactions and are only detected in specific environments, requiring validation through independent population or multi-environment experiments. The overlapping QTL intervals reported in multiple studies need to be combined with reference genome versions and linkage disequilibrium analysis to determine whether they are the same major effect locus or multiple closely linked independent loci. As a heterozygous tetraploid, *Brassica napus* has a large number of homologous copies in the A and C subgenomes, and homologous sites are prone to interference in localization signals. When interpreting, it is necessary to distinguish the homologous relationships between subgenomes. In addition, many loci exhibit pleiotropy and can simultaneously regulate multiple yield-related traits, requiring a balance of antagonistic relationships between traits in breeding utilization. Overall, only a few stable QTLs that have been independently validated can be developed as molecular markers and truly applied in rapeseed molecular breeding; A large number of candidate loci detected by GWAS and linkage localization still require further functional analysis.

## 5. Breeding, Biotechnology, and CRISPR Advances

First, radiation dose tests were conducted on the seeds. Seeds germinated at all doses, but seedling survival depended on seed moisture content at the time of irradiation: a dose of 350 Gy caused seedling death after soaking; a dose of 700 Gy caused seedling death after moistening (absorbing water for 1 h); and a dose of 2500 Gy caused seedling death after drying. Homozygous restorers exhibited extremely low seed setting rates. In the field, these plants produced short, curved pods, few seeds, and non-erect stems, contrasting sharply with the outwardly normal appearance of heterozygous fertile plants.

Genetic markers (developed via SNPs and high-throughput sequencing) were used to assess genetic function, gene mapping, and allele associations in rapeseed [185]. Because isozyme analysis indicated an alteration in the Pgi-2 pattern within the R2000 family, two PCR markers derived from the PGI sequence were tested in winter/spring rapeseed maintainer/restorer varieties, as well as in *Brassica oleracea* and *Chinese cabbage* (*B. rapa*) species. SG129 amplified two distinct bands in *Brassica napus*, with lengths of approximately 900 bp and 850 bp, respectively. The genome-specific PCR marker CP418 was designed to utilize the 5 bp polymorphisms between the two bands of SG129 in *Brassica napus* and was amplified using primers lower than CP418.

The SG129U primer amplified a 650 bp PCR band in a maintainer of *Brassica napus*, with a sequence strictly homologous to the *Brassica napus* EM:BH448336 sequence (Sequence 2) in diploid *Brassica* species. The BolJon marker showed a distinct 950 bp band in *Brassica napus* and a distinct 870 bp band in Chinese cabbage varieties. Five markers (four RAPD markers, AE19.700, AJ06.1100, O20.1380, M13.1420, and one specific PCR marker, IID12.1) located near introgression or substitution regions [186,187] were present in Drakkar but absent in R211 and Wesroona. Currently, MAS and transgenic breeding are commonly used methods in rapeseed breeding [188]. Genetic markers can be introduced for rapeseed breeding based on the dehydrin Protein (DHN) gene. MAS of rapeseed coat (*B. napus*) is an important step in improving the quality of rapeseed meal. Interestingly, the two regions BolJon and PGI revealed by PCR markers showed differences in homology among the tested rapeseed genotypes. In spring and winter rapeseed lines, the BolJon sequence in *Brassica rapa* was highly homologous and very close to that in the *Brassica rapa* variety (*Brassica rapa cv*.). The BolJon and CP418 markers in *Brassica oleracea* showed *Brassica oleracea* specificity in all backgrounds. The PGI sequence in *Brassica oleracea* showed higher homology to the *Brassica oleracea* variety (*Brassica rapa cv*.). The genome structure of *Arabidopsis thaliana* has been studied in detail [189], and comparative physical/genetic maps of *Arabidopsis thaliana* and *Brassica* have been constructed, mapping *Arabidopsis thaliana* marker sites to linkage groups based on AFLP, RFLP, EST, or SSR in *Brassica oleracea*, *Brassica napus*, or other *Brassica* species. By comparing recently obtained data [190,191,192,193,194], it was found that among various *Brassica* species, the most extensive breeding research on increasing seed oil content is on *Brassica napus*. The oil content of *Brassica napus* germplasm ranges from 35% to 52%. Notably, China recently bred a line with an oil content of approximately 65% through the aggregation of high oil content alleles [195].

This high oil content is not merely a single ideal trait, but a comprehensive reflection of a series of excellent agronomic traits and seed quality, including high yield. Over the past decade, plant breeders in Europe and Canada have continuously improved the seed oil content of rapeseed varieties. Compared to Canadian spring rapeseed, the increase in oil content in European winter rapeseed has been relatively less significant. This may be partly due to the longer breeding cycles required for winter rapeseed. Increased oil content, along with increased seed yield, does indeed improve oil yield per unit area. However, to date, the number of QTLs actually used for breeding is very limited. Selecting traits such as high oil content, thin seed coat, and large seeds can improve oil yield in *Brassica napus.* Some *Arabidopsis* homologs involved in lipid biosynthesis are located on QTLs related to seed oil content in *Brassica napus* [196]. Generally, there is no strong correlation between seed size and oil content. Seed oil is mainly composed of triacylglycerols (TAGs), which are formed by the esterification of three fatty acids onto a glycerol backbone. Although seed oil content in *Brassica* plants is controlled by many QTLs, numerous reports have successfully increased seed oil content in *Brassica napus* by regulating specific steps in fatty acid synthesis, glycerol ester assembly, or other carbon metabolism pathways [197].

Overexpression of the gene encoding *Arabidopsis* cytoplasmic acetyl-CoA carboxylase (ACCase) in plastids during seed development in *Brassica napus* represents an early success in increasing seed oil content in this oilseed crop [197]. Overexpression of type 1 DGAT in *Brassica napus* to increase seed oil content is another successful example [198]. With the continued growth in demand for commercial oil and the limited arable land area, metabolic engineering to increase seed oil content shows great potential. Therefore, silique traits and seed weight have long been considered important targets for genetic improvement in rapeseed. After removing redundant genes from different sources, a comprehensive list containing 1905 candidate genes related to silique and seed size was compiled. Currently, 151 gene-edited plants have targeted 84 candidate genes. These genes include 71 genes screened from transcriptome-wide association study (TWAS) data at different developmental stages of siliques, 11 genes from existing literature, and 2 genes specifically expressed in the siliques of *Brassica napus*. The genes used in this study the CRISPR/Cas9 system to generate various editing types in candidate genes, including single-base insertions or deletions, base substitutions, large-fragment insertions or deletions, and mutations with multiple editing methods occurring at the same location. Using the pRGEB32-BnU6C01 vector backbone, we constructed a CRISPR/Cas9 mutant library targeting candidate genes in *Brassica napus* related to silique and seed size.

Using an integrated breeding platform, previous studies purified and screened a subset of gene-edited individuals was purified and screened, ultimately obtaining 10 homozygous transgenic lines. These lines target homologous genes of *Brassica napus*, including *AT2G36450*, *AT5G57790*, *AT5G47330*, *AT3G52115*, *AT5G52050*, *AT1G24150*, *AT5G52600*, *AT2G36450*, and *AT2G43120*. However, due to safety concerns, transgenic rapeseed cannot be widely applied [199]. In recent years, precision gene-editing technologies, represented by CRISPR/Cas9, have developed rapidly, achieving significant progress in major crops such as rice and wheat. This demonstrates immense application value in rapeseed breeding and improvement, and will accelerate directional variation and evolution in breeding. As of 2020, numerous publications have reported the successful application of CRISPR/Cas9 technology in rapeseed breeding [200,201,202,203,204], indicating that the application of this technology in rapeseed is becoming increasingly mature and has been widely used in the construction and genetic improvement of rapeseed germplasm resources. CRISPR/Cas9 technology has also become an important tool for analyzing gene function and molecular mechanisms in *Brassica napus.*

Previous studies have shown that using CRISPR/Cas9 technology to improve target traits is entirely feasible [205]. However, as a novel allotetraploid (AACC) species [206], *Brassica napus* exhibits a significantly increased copy number of related genes compared to its ancestors and other older varieties. Most genes in the *Brassica napus* genome have multiple copies, resulting in severe functional redundancy, which hinders the progress of *Brassica napus* genomics research [207]. CRISPR/Cas9 technology relies on gene transformation techniques. Therefore, establishing a stable and efficient gene transformation system for *Brassica napus* is crucial [159]. Furthermore, (CRISPR/Cas9) CRISPR/Cas9 introduction methods include the gene gun method, protoplast transformation method, pollen channel method, and electrical stimulation method [208]. High specificity and precision in gene editing are essential in gene editing and breeding processes. To address the lack of target sites, researchers have employed various strategies to improve the specificity of gene editing, such as dual Cas9 cleavage enzymes [209], sgRNA shortening [210], and FokI-dCas9 fusions [211]. Currently, there is no specific promoter design for the CRISPR/Cas9 system in *Brassica napus*. However, the Cpf1 editing system [211] developed by Chinese-American scientist Feng Zhang has greatly simplified the complex multi-gene editing process. Applying it to the breeding of *Brassica napus* is expected to yield superior varieties. The application of CRISPR/Cas9 gene editing technology in yield-related traits has mainly focused on pod cracking resistance and multilocular pods. However, due to its complexity, research progress on genes related to traits such as seed weight and oil content has been relatively slow. As shown in Table 4, the biotechnology methods and CRISPR-based genome editing techniques for improving seed traits in *Brassica napus* are presented.

It is widely believed that the absence of petals is significant for improving the photosynthetic efficiency and disease resistance of *Brassica napus*, but there are currently no literature reports on the application of gene-editing technology in this field. High quality is another important goal in oilseed crop breeding. Studies have found that gene-edited *Brassica napus* plants (*BnaSDG8* and *BnaRGA*) flower more than 40 days earlier than ordinary plants. In rapeseed, the expression of the *BnaWRKKY11* and *BnaWRKKY70* genes differed after inoculation with *Sclerotinia sclerotiorum* (Lib.) de Bary. Compared with black-seeded rapeseed, yellow-seeded rapeseed has advantages such as a thinner seed coat, higher oil and protein content in the oilseed cake, and better quality. Furthermore, yellow-seeded rapeseed has great potential for application in hybridization breeding. Using the selected primer combinations E32M47, E32M60, and E33M62, a total of 467 polymorphic AFLP bands were scored in *B. napus* RS lines and spring rapeseed varieties.

**Figure 6:**
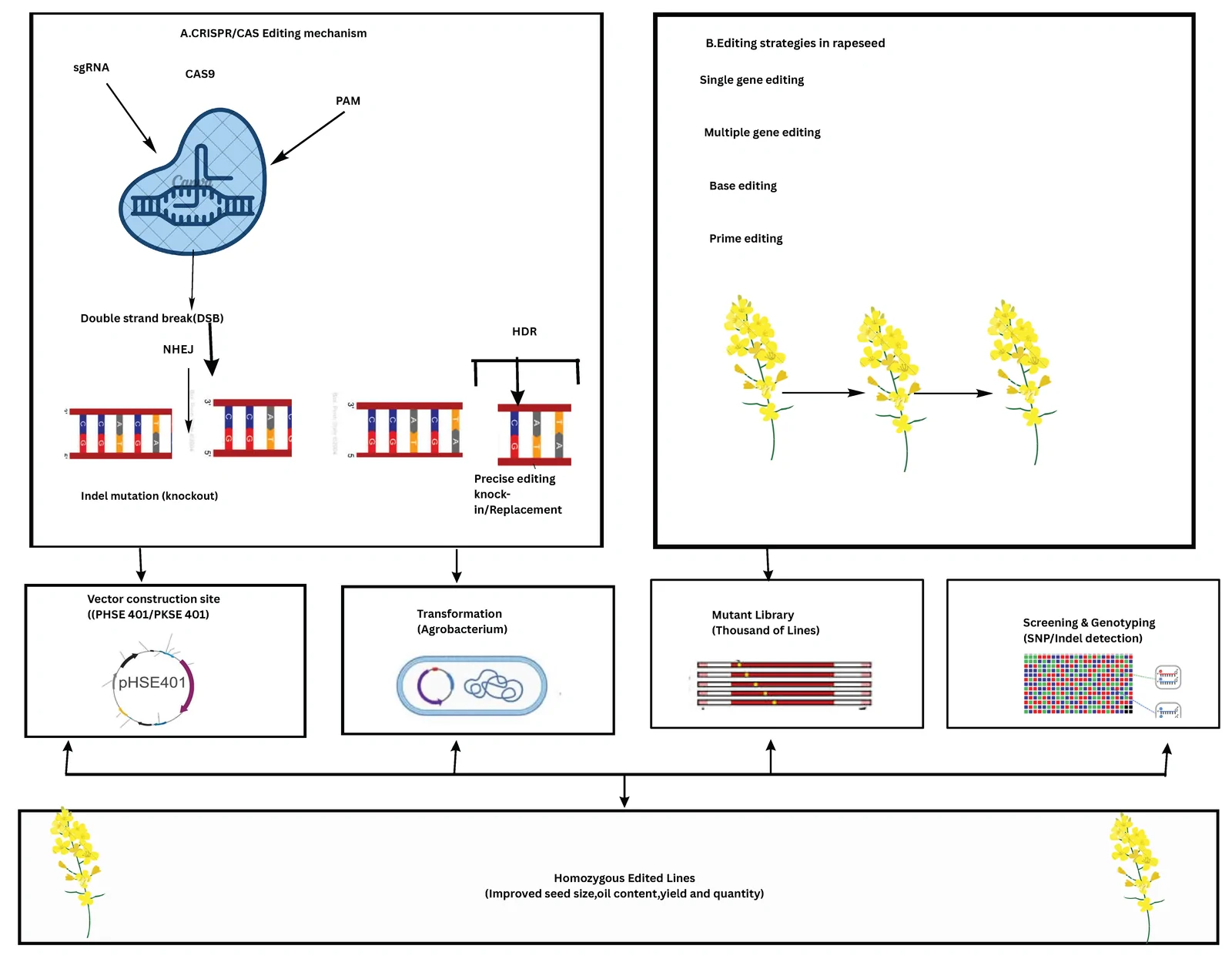
The modern breeding and biotechnological methods used to improve seed size, yield, and quality traits in *Brassica napus*. The CRISPR/Cas genome editing system enables precise modification of target genes through double-strand DNA cutting followed by repair using non-homologous end joining (NHEJ) or homology-directed repair (HDR). Various editing strategies, including single-gene editing, multiplex editing, and base editing, contribute to the development of desired traits. Combining genome editing with traditional breeding, gene transformation, mutant screening, and trait validation can accelerate the identification and introduction of beneficial alleles. These methods collectively improve breeding efficiency and support the development of improved rapeseed varieties with superior agronomic traits and seed quality.

The enormous potential of recombination in rapeseed breeding will make germplasm resource conservation and the rational utilization of diploid parents particularly important in the future. Clearly, the establishment of new gene banks based on novel *Brassica napus* is limited by its poor agronomic traits and seed quality (++); therefore, this approach must be viewed from a longer-term perspective. However, the discovery of low erucic acid mutants in *Brassica oleracea* germplasm resources [212], and the breeding of high erucic acid rapeseed varieties through interspecific hybridization with high-quality grade 0 or 00 *Chinese cabbage* (*B. rapa*) genotypes will pave the way for utilizing these new rapeseed materials as genetic resources to improve rapeseed quality and yield. Especially in China, significant progress has been made in the research and breeding of rapeseed oil content in recent years. Combining genome editing with traditional breeding, gene transformation, mutant screening, and trait validation can accelerate the identification and introduction of beneficial alleles, as shown in Figure 6.

CRISPR/Cas-based genome editing biotechnology provides powerful tools to dissect the regulatory mechanisms of seed size and facilitate targeted yield improvement in allotetraploid *Brassica napus*, yet its application in rapeseed still faces unique biological and technical challenges specific to polyploid species. As *Brassica napus* contains A and C subgenomes, many genes have multiple homoeologous copies with widespread functional redundancy and dosage effects. Knocking out only partial homeologs often fails to produce obvious phenotypic changes, and multiplex editing of several copies is frequently required to fully expose gene function, imposing higher requirements for sgRNA design and vector construction. Genetic transformation and plant regeneration in rapeseed suffer from strong genotype dependence and low regeneration efficiency; chimeric and heterozygous editing events commonly occur in T_0_ plants, complicating the screening of stable homozygous edited lines. Targeted editing of seed-development genes frequently induces pleiotropic effects on organ growth and fertility; enhanced seed size may be accompanied by abnormal silique development and reduced seed set. Compared with complete gene knockout that leads to total loss-of-function, promoter editing enables fine-tuning of gene expression while retaining basal gene activity, which is better suited for quantitative traits such as seed size and provides milder genetic manipulation strategies. A key concern is the inherent trade-off between seed size and seed number after editing seed-regulatory genes: improvement in one yield component often compromises the other and limits final grain yield. Most current CRISPR studies are limited to phenotypic characterization under greenhouse or pot conditions, with insufficient multi-site field validation. In addition, the commercial deployment of edited rapeseed materials is constrained by biosafety and regulatory frameworks. Collectively, CRISPR holds great potential for dissecting seed-size regulatory genes in rapeseed, but this technology is not mature or widely deployed in practical rapeseed breeding. Future research should prioritize multi-environment field evaluation and deploy functionally validated favorable alleles into elite germplasm, translating fundamental genome-editing findings into breeding-usable genetic resources.

Although CRISPR/Cas gene-editing technology provides a powerful research tool for analyzing the function of seed size regulatory genes and targeted improvement of grain weight traits in *Brassica napus*, there are still multiple practical bottlenecks in the transition of this technology from laboratory concept validation to breeding applications. As a heterozygous tetraploid, *Brassica napus* widely suffers from functional redundancy of homologous genes. Single-copy editing often fails to produce obvious phenotypes and requires simultaneous editing of multiple homologous copies, increasing the difficulty of vector construction and target design. At the same time, target genes generally exhibit pleiotropy, and editing and improving seed size may lead to changes in related traits such as pod development, plant type, or oil content, resulting in a yield trade-off effect. In addition, there are significant differences in genetic transformation efficiency among different genotypes of *Brassica napus*, and in vitro plant regeneration is difficult, which limits the scale of editing material creation; CRISPR editing also requires systematic off-target effect detection and evaluation to eliminate the risk of unexpected genomic mutations. The excellent traits obtained through gene editing still need to be identified in multiple field environments over many years to verify their environmental stability and avoid laboratory phenotypes that cannot be stably reproduced in the field. In addition, gene-edited crops also face strict biosafety and regulatory restrictions. Different countries have different regulatory policies for gene-edited materials, which restrict the variety registration and industrial promotion of edited materials. Overall, CRISPR technology has great potential in molecular breeding of rapeseed seed traits, but it still needs to overcome the technical bottlenecks and regulatory challenges mentioned above in order to truly serve the cultivation of high-yield rapeseed new varieties.

**Table 4:** Biotechnological Approaches and CRISPR-Based Genome Editing for Seed Trait Improvement in Rapeseed (*Brassica napus*).

Technology	Target Genes/Loci	Evidence Type	Purpose	Key Outcome	Significance in Breeding	Main References
CRISPR/Cas9	BnaEOD3	1	Increase seed size	Larger seeds and modified silique morphology	Enables precision genome editing for seed trait improvement	[99]
CRISPR/Cas9	BnaSDG8	1	Manipulate flowering time	Flowering accelerated by 40 days	Improves crop environmental adaptation and cropping system flexibility	[213]
Gene Editing Library	84 candidate genes	1	High-throughput functional validation	151 independent edited mutant lines generated	Supports large-scale screening of genes controlling agronomic traits	[214]
SNP Marker Technology	Yield-related loci	2	Marker-assisted selection	High-density genetic maps constructed	Accelerates conventional breeding by marker-assisted genotype screening	[215]
DArT Mapping	Oil and yield trait loci	2	Genome-wide linkage analysis	139 trait-associated genetic intervals identified	Facilitates germplasm genetic diversity evaluation and QTL mining	[216]
Transgenic ACCase Overexpression	Lipid biosynthesis genes	1	Increase seed oil content	Enhanced triacylglycerol (TAG) accumulation in seeds	Metabolic engineering strategy to boost oil yield	[217]
DGAT Overexpression	TAG assembly pathway genes	1	Improve oil biosynthesis	Elevated seed oil concentration	Targeted metabolic engineering for oil quality and yield improvement	[198]

Note: Evidence Type (same as Table 1).

## 6. Environmental and Physiological Effects

**Figure 7:**
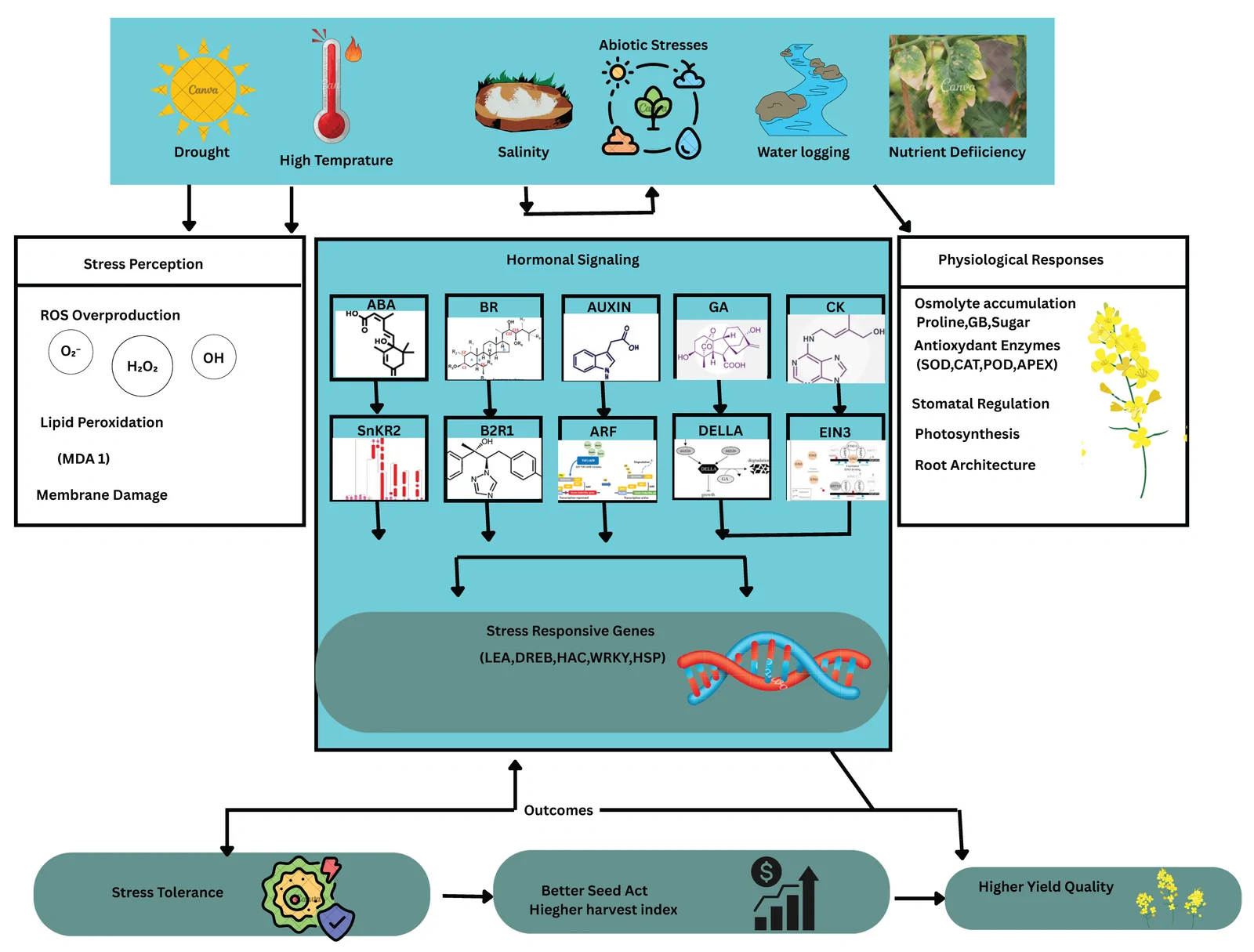
The effects of major abiotic stresses such as high temperature, drought, salinity, and waterlogging on seed development. Stress perception triggers the accumulation of reactive oxygen species (ROS), lipid peroxidation, activation of hormone signaling pathways (abscisic acid, brassinosteroids, and auxins), and expression of stress-responsive genes. These responses lead to a series of physiological and biochemical changes, such as the accumulation of osmotic regulators (proline, glycine betaine, and sugars), enhanced activity of antioxidant enzymes (superoxide dismutase, catalase, peroxidase, and ascorbate peroxidase), stomatal regulation, and alterations in photosynthesis. These mechanisms collectively determine plant stress tolerance and ultimately influence seed yield and productivity.

Rapeseed is an annual plant. Winter-growing, semi-winter-growing, and spring-growing rapeseed varieties differ in their cold and drought tolerance, thus requiring different growing conditions. Winter-growing rapeseed thrives in relatively high humidity and low temperatures. Rapeseed cultivation requires well-drained soil with a pH between 5.5 and 8.5 for optimal growth. The quantitative trait of rapeseed oil content is controlled by multiple genes and influenced by environmental factors. Some studies have shown that most QTL loci detected in the same population under different environments are environment-specific, indicating that adaptation to different environmental factors plays an important role in breeding high-oil-content varieties carrying strong alleles [218]. Temperature and light are the two major environmental factors affecting rapeseed oil yield [136,219,220]. High temperatures during rapeseed maturity are unfavorable for nutrient accumulation and conversion to seeds [136]. As shown in Figure 7, the effects of major abiotic stresses such as high temperature, drought, salinization, and flooding on seed development are demonstrated.

Rapeseed seed weight and oil content are significantly influenced by environmental factors and cultivation methods, with temperature during seed maturity playing a crucial role [221,222,223,224]. However, QTLs are sensitive to the environment, and the molecular mechanisms of QTL-environmental interactions remain poorly understood. Seed development is a key process in the life cycle of higher plants, comprising morphogenesis and maturation. Ref. [225] also observed a significant effect of environmental factors on oil content variation.

The oil content of sunflower seeds is highly sensitive to environmental conditions such as temperature and radiation during the seed-filling stage [91]. Similar environmental influences (temperature, rainfall) have been observed in rapeseed [226,227]. Oil synthesis in plant seeds is a complex physiological and biochemical process involving two steps: fatty acid synthesis (FAS) and triglyceride (TAG) accumulation. Physiological and biochemical studies have shown that resistance to sclerotinia stem rot and lodging resistance are important factors affecting rapeseed oil content [228].

Regulatory factors such as LEC2, WRI1, FUS3, MYB30, and ABI3 are active during early seed development. Some of these factors, including LEC2, WRI1, FUS3, and FUS3, remain active during seed maturity, indicating that they play a major regulatory role during this period. OLEO1 [229,230] is present in oil bodies involved in seed lipid accumulation. Generally, temperature has a significant impact on seed oil content and fatty acid composition. Higher temperatures at seed maturity result in lower seed oil content and yields of unsaturated fatty acids (such as linoleic acid (C18:3)). High temperatures at seed maturity are a significant limiting factor for yield in many rapeseed-producing areas, such as the middle and lower reaches of the Yangtze River in China. Previous studies noted that *BnABA3* (an important regulator in ABA signaling) is upregulated at high temperatures. Because ABA plays a crucial role in seed maturation and dormancy, this may lead to accelerated seed maturation and reduced accumulation of seed storage substances under high-temperature conditions. A dynamic regulatory map was developed to understand the responses among stress response target genes and predicted the dominant roles of *LEC1*, *LEC2*, *WRI1*, *FUS3*, *MYB30*, and *ABI3* in controlling seed formation and potential interactions in *Brassica napus*. However, it is noteworthy that in 2011 and 2013, even after a two-year interval between rapeseed crops (one year in 2011), free-growing rapeseed remained the dominant species. Rapeseed seeds are known to have secondary dormancy [231] and can remain dormant for several years in Western Canadian planting systems [232]. The main reason for the yield declines when rapeseed is planted on its own crop residues is the increased inoculation with pathogens [233,234,235]. There was no significant interaction between crop rotation frequency and rapeseed variety for rapeseed yield. In all years, yield increased linearly and significantly with increasing intervals from 0 to 2 years between rapeseed crops (*p* ≤ 0.002). In 2011, the yield effect exhibited a quadratic trend (*p* = 0.074), indicating that most of the yield increase occurred when wheat was intercropped with rapeseed for one year. For each year of increase in crop rotation diversity, the linear slope coefficient of rapeseed yield varied between 0.20 and 0.36 Mg ha^−1^. These results are consistent with many other studies that have shown that increased crop rotation diversity can improve rapeseed yield [236,237,238,239,240,241,242].

Early weed control mitigated weed-induced yield losses in rapeseed [243,244,245]. PLS analysis enabled us to identify site conditions associated with high rapeseed yields [246,247]. Drought is one of the major abiotic stresses that can have devastating effects on crop growth, development, and yield [248,249,250,251]. Water deficit also disrupts mineral and water uptake, assimilate synthesis, and distribution, leading to severe yield reductions [252,253]. *Brassica* plants are primarily affected by drought stress because they are mainly grown in arid and semi-arid regions [254,255,256]. Rapid germination and successful seedling establishment are crucial for normal growth and profitable rapeseed production [257,258]. Decreased enzyme activity and photosynthetic rate lead to a significant reduction in rapeseed biomass yield [259]. Compared with healthy plants unaffected by reactive oxygen species (ROS), rapeseed damaged by ROS showed a significant decrease in yield [260,261], while ROS also increases the production of hydrogen peroxide (H_2_O_2_), thereby promoting lipid peroxidation [262]. During the flowering period, the number of siliques and seeds in rapeseed decreased under stress conditions [263]. The stress-tolerant rapeseed variety (P287) exhibited a higher content of osmotic regulators; however, the content of osmotic regulators may vary depending on the specific tissue and the physiological characteristics of *Brassica* species and varieties [264]. Various environmental stresses affect endogenous stress levels and functional homeostasis, which are central to regulation and tolerance mechanisms [265]. In rapeseed, gene regulatory networks have revealed components in the signaling of various hormones, including abscisic acid (ABA), brassinosteroids (BRs), and auxins, which play crucial roles in stress tolerance [266]. Similarly, the genes BnNAC485, BnSIP1-1, and BnABI5 expressed in transgenic *Brassica napus* L. are associated with resistance to abiotic environmental stress [267]. Furthermore, researchers have identified differentially expressed genes (DEGs) in various *Brassica napus* varieties, such as ERD15, LEA14, and RAB18, which are associated with abiotic stress tolerance [268]. GWAS have been used to elucidate the genetic control mechanisms of rapeseed seed weight, oil quality, and seedling vigor.

Transfection of the *EOD3* gene yielded transgenic *Brassica napus* plants that exhibited superior growth performance under stress compared to the wild type [269]. Exogenous application of glycine betaine (GB) improved drought tolerance in rapeseed by increasing chlorophyll content, seed yield, carbohydrate content, proline content, antioxidant activity, and root length, while decreasing malondialdehyde (MDA) and hydrogen peroxide (H_2_O_2_) levels [270]. Plant growth regulators (PGRs) are small-dose organic substances used to regulate various physiological processes in plants, thereby improving plant development and enhancing their stress resistance [271]. Seed pretreatment with different substances can improve osmotic regulation, photosynthetic efficiency, physiological activity, and reactive oxygen species (ROS) scavenging capacity. Compared with control plants under drought stress, seed pretreatment significantly increased the number of seeds per pod, seed weight, and seed yield [255,272,273]. Because little is known about the genotype-environment interaction in *Brassica napus*, QTLs identified under specific conditions may not function effectively under other conditions. Some studies have shown that insufficient water is a major limiting factor affecting seed germination [274,275]. Temperature is a key environmental factor affecting seed germination [276,277].

Some studies have shown that seed germination rate increases linearly with increasing temperature until a suitable temperature is reached, after which it decreases linearly as the temperature exceeds this limit [278]. Low temperatures reduce enzyme activity and slow nutrient mobilization, thereby limiting the metabolic processes required for seed germination and development [279]. Good seed germination rates allow for higher planting density, which is crucial for the yield and stability of directly sown rapeseed [280]. Generally, increasing planting density increases seed yield. Furthermore, abiotic stresses such as drought, high temperature, and salinity negatively impact plant growth, leading to decreased yield and crop quality [281]. Additionally, rapeseed seeds are small and prone to dehydration under severe abiotic stress. In scientific research aimed at improving seed germination and crop emergence rates, pre-sowing seed treatment and agronomical measures have received increasing attention [282,283,284].

Literature indicates that differences in seed size can lead to variations in seed-water relationships, thereby affecting seed emergence ability at different sowing depths [285] and resistance to water stress [286,287,288]. Furthermore, numerous studies have documented the minimum water potential required to induce seed imbibition [275,289]. Excessive waterlogging depletes seeds of available oxygen, a crucial factor for seed germination [290]. Seedling length increases significantly with increasing moisture content, reaches a maximum, and then decreases significantly with further increases in moisture. Under ideal conditions, seedlings grow rapidly, consistent with previous findings [289]. As shown in Table 5, the environmental and physiological factors that affect the development, seed size, and yield of rapeseed seeds are comprehensively elucidated.

Environmental conditions can alter the size of *Brassica napus* seeds by regulating physiological processes during key stages of seed development, and this response exhibits significant genotype-by-environment interactions. The seed filling stage is the core developmental window period that determines the final grain size. During this stage, temperature, water availability, nutrient supply, and population planting density can regulate the supply of photosynthetic products from source organs, the transport and storage of assimilates from sink organs, and change the source-sink balance, thereby affecting cell proliferation and grain filling processes, ultimately shaping the seed size phenotype. There are differences in the response of different genotypes to environmental signals, and the regulation direction and amplitude of the same environmental stress on different germplasm seed sizes are not consistent. Only environmental factors applied during the critical window of seed development will significantly alter the final seed weight. If the internal mechanism of its effect on grain development is not elucidated, it is not within the scope of this section if it deviates from the study of seed development timing, generalized drought tolerance throughout the entire growth period, and intercropping systems. Analyzing how environmental factors regulate seed size during specific developmental windows and uncovering the genetic basis of genotype-environment interactions will provide theoretical support for targeted regulation of rapeseed seed size and yield improvement in different ecological regions.

## 7. Molecular Pathways and Seed Quality Traits

Increased marker density improved the localization resolution of QTL for seed color and fiber content. Flanking marker sequence prediction indicated that Bna.CCR1 was located in the major QTL for seed fiber content in the ExV8-DH population with a dark seed genetic background, consistent with the findings of [147] in yellow-seeded *Brassica napus*. ATPase *Bra039228* was first identified as a highly promising candidate gene for both location and function, potentially significantly influencing seed color variation in this *Brassica napus* population. The major QTL for seed color (which also significantly affected seed coat thickness and fiber content, suggesting pleiotropic effects) was decomposed into the aforementioned minor sub-QTLs and a major sub-QTL with a particularly significant impact on seed color (partial R^2^ = 32.9%). The SSR marker Ni4D09 consistently remained near the peak of the major QTL controlling both seed color and seed fiber traits.

Using markers developed from *Brassica rapa* sequence data, the genetic map of YE2-DH was enriched in the major QTL region controlling seed color on chromosome A9. The genetic and/or physical locations of the SSR markers KBrH092O19 and KBrB025M01, flanking the QTL controlling fiber compounds in both ExV8-DH and YE2-DH, were completely consistent with the major QTLs controlling seed color and ADL content previously identified in completely unrelated Chinese rapeseed hybrids [147]. Since the 1970s, the genetic diversity of superior breeding materials has further decreased due to continuous selection for seed quality traits, particularly through the introduction of double-low (00) seed quality (zero erucic acid, low glucosinolate content) traits (mainly derived from two superior varieties as donors) [298]. This is consistent with previous findings in rapeseed, where quantitative genetic studies have shown that additive gene effects dominate in controlling seed oil content [299,300,301]. QTL mapping results by Zhao indicated that additive effects are the main factor contributing to variation in oil content. The results of [302] showed that most variations in rapeseed oil content are unrelated to variations in developmental traits. Previous studies have confirmed that multiple QTL control oil content in rapeseed, some of which may be ubiquitous across different genetic backgrounds, while others are genetically background-specific. Genetic transformation studies have confirmed that these genes can increase seed oil content to varying degrees; for example, transgenic *Brassica napus* expressing cytoplasmic acetyl-CoA carboxylase (ACCase) in plastids showed a relative 5% increase in seed oil content [303]. Furthermore, overexpression of the nasturtium *DGAT1* (*TmDGAT1*) gene in erucic acid-rich *Brassica napus* also resulted in a relative increase in seed oil content of 11–30% [304].

**Figure 8:**
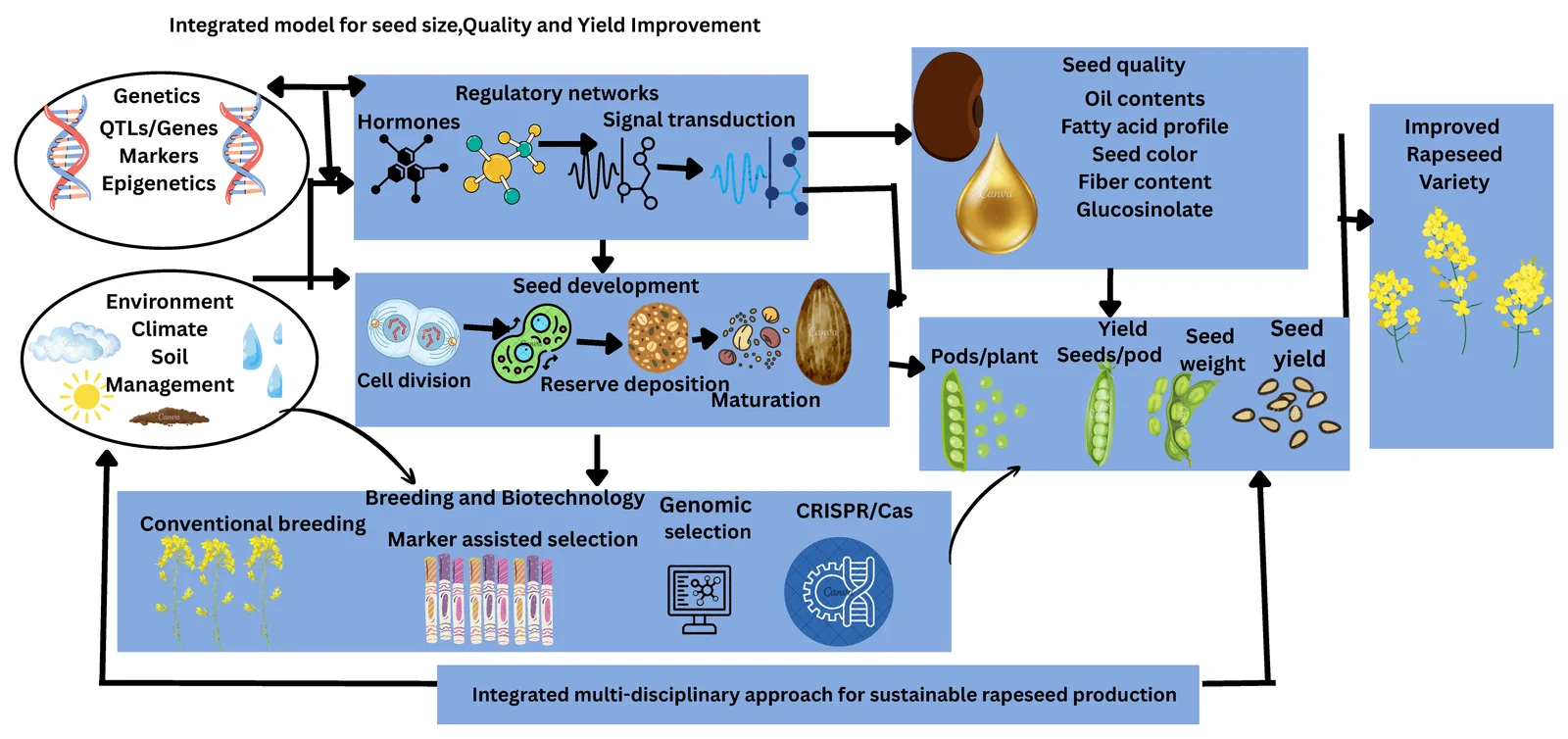
An integrated model for the improvement of rapeseed seed size, quality, and yield. This model synthesizes genetic and epigenetic regulation, QTLs/genes, molecular markers, hormone signaling, environmental and management factors, and seed development processes—including cell division, storage substance accumulation, and maturation—while encompassing traits such as oil content, fatty acid composition, seed color, fiber content, and glucosinolate profiles.

**Figure 9:**
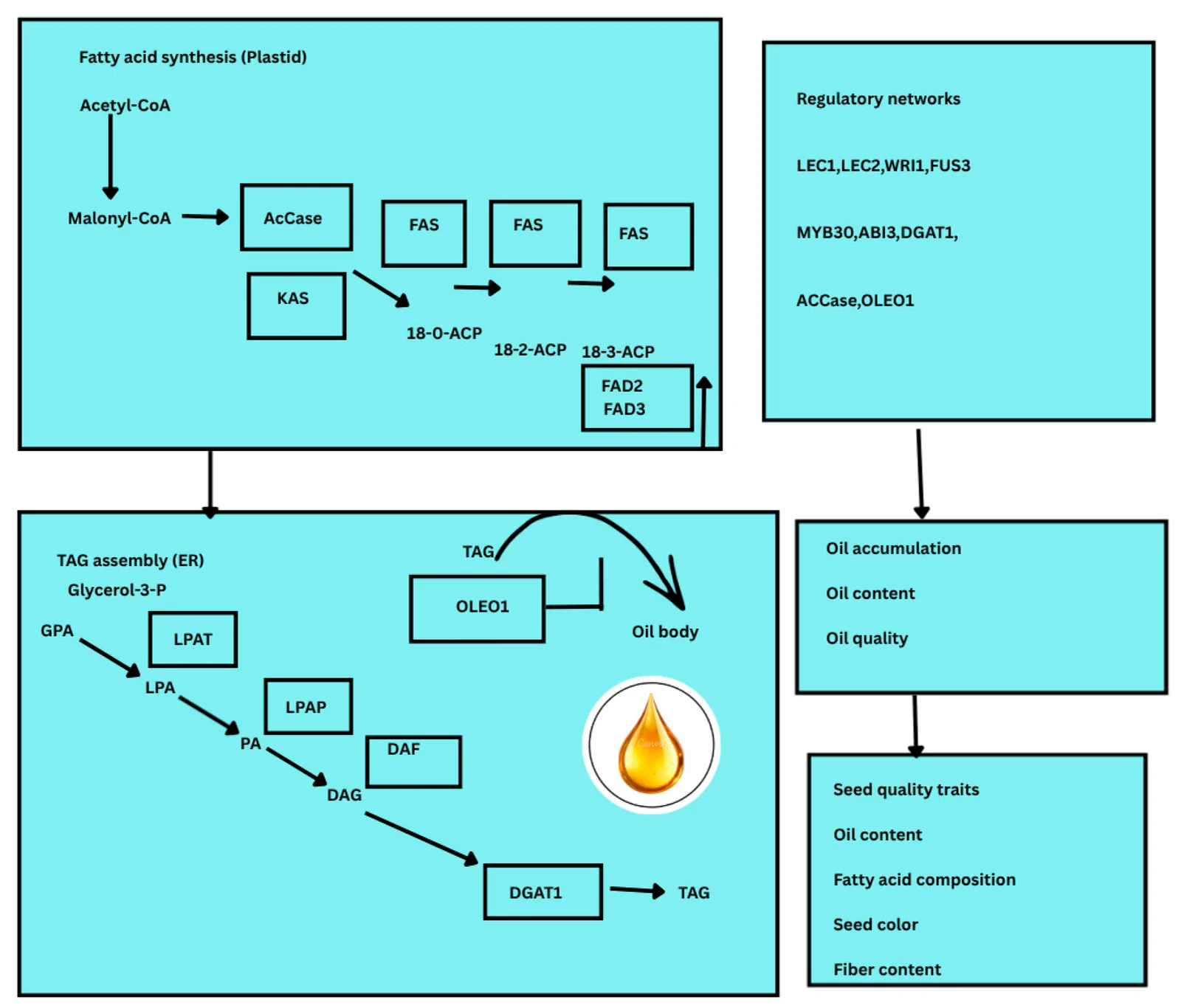
This diagram illustrates the key biochemical and genetic mechanisms underlying seed oil biosynthesis and quality determination. Fatty acid synthesis in the plastids is mediated by enzymes such as ACCase, KAS, FAS, FAD2, and FAD3, while triglyceride (TAG) assembly occurs in the endoplasmic reticulum via the LPAT, PAP, DGAT1, and OLEO1 pathways. Regulatory genes, including LEC1, LEC2, WRI1, FUS3, ABI3, MYB30, ABI5, DGAT1, ACCase, and OLEO1, coordinate oil accumulation, oil content, and oil quality. These molecular processes collectively influence important rapeseed quality traits such as oil content, fatty acid composition, seed color, and fiber content.

Ref. [305] used 116 molecular markers associated with oil content. Their study showed that approximately 50% of the markers were related to oil content. Ref. [306] also conducted a similar study. Since seed oil content and seed yield traits are closely related, elucidating the relationship between various traits and oil content is crucial for breeding high-oil-content varieties. As shown in Figure 8, a strategy for identifying candidate genes related to seed size, oil yield, and other agronomic traits is presented. A study on the correlation between seed oil content and several major yield traits in 16 varieties showed that the order of positive contribution was: number of pods > lodging rate > thousand-seed weight > yield > main inflorescence fruitlessness rate > number of siliques [307]. Another study showed that rapeseed oil content was significantly positively correlated with the number of seeds per silique (r = 0.421), while it was only weakly positively correlated with the weight of a single seed (r = 0.172) [302]. In 2007, Dr. Fu Tingdong of Huazhong Agricultural University, through hybridization and a series of selection processes, bred rapeseed varieties with high oil content (up to 54.72%) and low oil content [308]. Using similar techniques, ref. [309] of the Jiangsu Academy of Agricultural Sciences used a high oil content population as the selection population to conduct positive screening of seed oil content, obtaining the high oil content variety HOC3 with an oil content of 54.52%. The combination of genetic engineering, molecular marker technology, and traditional targeted selection strategies will be the development trend of high oil content rapeseed breeding. As shown in Figure 9, the key biochemical and genetic mechanisms involved in seed oil biosynthesis and quality determination are elaborated in detail.

## 8. Genome Evolution, Diversity, and Breeding Resources

**Figure 10:**
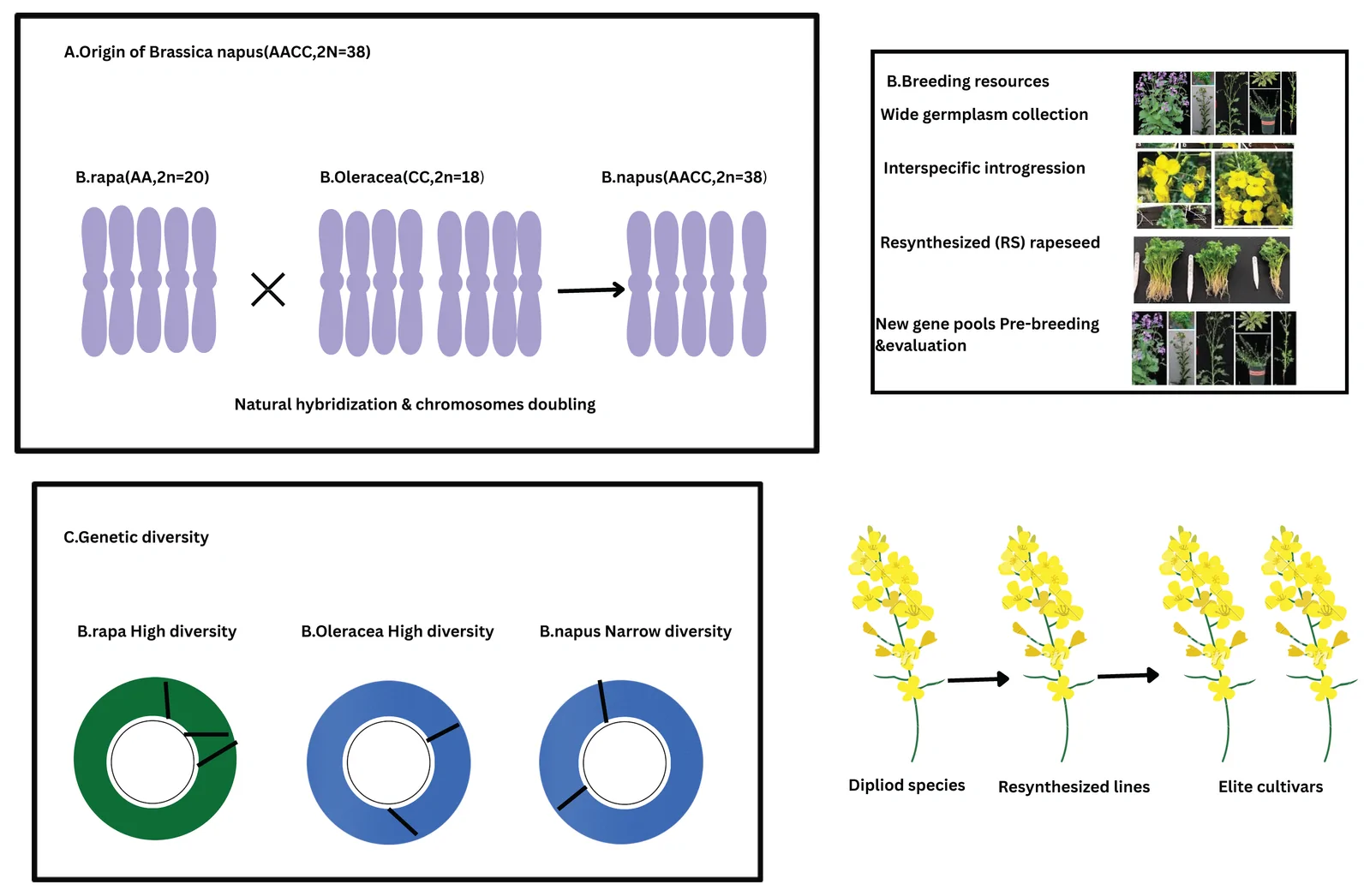
The evolutionary origin of rapeseed, showing how its ancestral species, *Brassica rapa* (AA, 2n = 20) and *Brassica oleracea* (CC, 2n = 18), produced the allotetraploid species *Brassica napus* (AACC, 2n = 38) through natural hybridization and chromosome doubling. The figure also highlights available breeding resources, including wild germplasm, interspecific introgression lines, recombinant rapeseed populations, and novel gene pools. It depicts the genetic diversity between ancestral species and cultivated rapeseed, emphasizing their importance in broadening the genetic base, improving yield and quality traits, and enhancing resistance to biotic and abiotic stresses.

Many important crops, such as wheat (*Triticum aestivum*), cotton (*Gossypium hirsutum*), and rapeseed (*Brassica napus*), have been confirmed as allopolyploids, composed of combinations of two or more distinct genomes [17]. The genetic diversity of some naturally occurring allopolyploids is lower than that of their parent species [310,311,312], leading us to speculate that a few variants in the parent species may contribute to the formation of these allopolyploids. To investigate the genetic structure of the *Brassica* genus, two molecular markers-SSR and AFLP-were used to perform a genome-wide genetic structure analysis of 49 germplasm resources representing natural varieties of *Brassica napus*, *Brassica rapa*, and *Brassica oleracea* cell populations. Findings support previous findings that rapeseed originated more than once [298,313], and that the C subgenome of rapeseed is associated with cultivated cabbage (*B. oleracea*) [313,314,315,316] or mountain cabbage (*B. montana*) [313]. As shown in Figure 10, the evolutionary origin of rapeseed is demonstrated, revealing how its ancestral species, *Brassica rapa* (AA, 2n = 20) and *Brassica oleracea* (CC, 2n = 18), produced the allotetraploid species *Brassica napus* (AACC, 2n = 38).

None of the virtual allopolyploid lines generated in this study perfectly matched natural *Brassica napus*. Similar results were observed in both natural *Brassica napus* and synthetic allopolyploids [310,317]. Natural *Brassica napus* germplasm from three different gene banks [310,318,319] clustered with virtual *Brassica napus* lines and parental species in principal component analysis. During the breeding process, frequent introgression from *Brassica rapa* (*B. rapa*) into *Brassica napus* has led to significant variation in the A subgenome of modern *Brassica napus* [310]. Among the three *Brassica* species tested, *Brassica oleracea* showed the highest genetic diversity in its cell populations, with an average genetic distance of 0.59 using SSR markers and 0.32 using AFLP markers. This was followed by *Brassica rapa* (0.33 using SSR markers and 0.25 using AFLP markers), and finally *Brassica napus* (0.24 using SSR markers and 0.13 using AFLP markers).

Notably, the ancestor of natural rapeseed corresponds to *Brassica rapa* and group C-I, supporting the view that rapeseed originated from *Brassica rapa* and *Brassica napus*. This indicates that natural rapeseed exhibits less variation compared to its parent species, thus holding great potential for expanding its genetic base using parent species. Currently, the genetic base of breeding materials is relatively narrow. Recombinant (RS) rapeseed genotypes bred through interspecific hybridization between different *Brassica napus* (*Brassica rapa*) and *Brassica oleracea* genotypes hold promise for significantly increasing the available gene pool and providing important basic germplasm resources for further improvement of seed yield, seed quality traits, and disease and pest resistance. *Brassica napus* is a relatively young species, originating in a limited geographical area and arising from natural hybridization between a small number of *Brassica napus* and *Brassica oleracea* genotypes. Therefore, the genetic variation of this important oilseed crop in many traits with breeding value is limited.

The potential benefits of recombinant technology in rapeseed breeding will make future efforts in germplasm resource conservation and the rational utilization of diploid parents even more important. Clearly, the construction of new gene banks based on artificially synthesized *Brassica napus* is limited by its poor agronomic traits and seed quality (++); therefore, this method must be viewed from a longer-term perspective. With the application of advanced breeding and production technologies, in the near future, the oil yield and quality of rapeseed varieties will be significantly improved, and more varieties with excellent traits, especially those that are early-maturing, high-yielding, resistant to biotic and abiotic stresses, and suitable for mechanized harvesting, will be developed. Functional genomics, as a supporting technology for molecular breeding, has become a key focus of rapeseed research in China over the past decade. China has made significant progress in identifying functional genes related to important traits such as yield, seed oil content, fertility regulation, disease and pest resistance, abiotic stress resistance, nutrient use efficiency, and pod cracking resistance.

In 2015, Chinese researchers identified genes that regulate seed weight (*BnaA9.ARF18*) and the number of seeds per pod (*BnaC9.SMG7b*) [96]. Transcription factors *LEC1* (foliate cotyledon 1) and *WRI1* (wrinkled 1) have been shown to be involved in the regulation of glycolysis, ultimately promoting the accumulation of oil in seeds. Maternal organs, including the pod wall and seed coat, have a significant impact on the oil content of rapeseed seeds [320]. In China, various marker technologies have been developed for rapeseed research, such as SRAPs. Markers linked to multiple major genes or QTLs related to important traits in rapeseed improvement programs have been successfully applied to marker-assisted breeding from 2005 to 2014; a total of 215 *Brassica napus* varieties were registered in China, including 210 winter-type varieties and 5 spring-type varieties. Multiple studies have shown that rapeseed is more likely to achieve high yields under high-density planting conditions.

## 9. Conclusions

**Figure 11:**
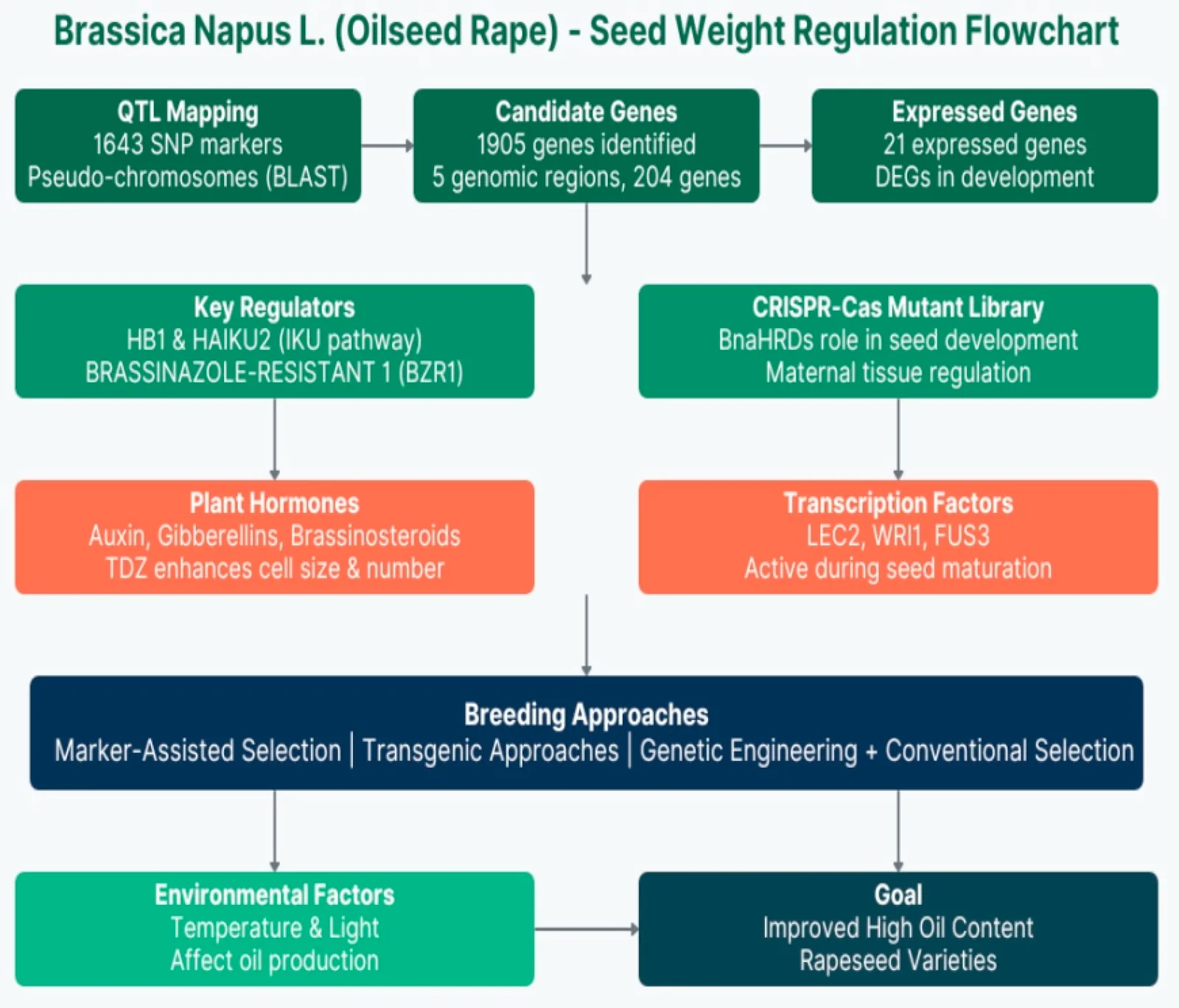
*Brassica napus* L.(Oilseed rape)-Seed weight regulation Flowchart.

Oil content and seed weight in *Brassica napus* are complex quantitative traits regulated by multiple hormonal, environmental, and genetic factors. Rapeseed protein is a nutrient-rich and highly functional plant protein with enormous potential for food applications. Advanced processing technologies can overcome current limitations and expand the application of rapeseed protein in sustainable foods and industrial products [321]. Recent advancements in QTL mapping, transcriptome analysis, and genome editing technologies have identified numerous candidate genes and regulatory pathways involved in seed development, including hormone signaling, transcriptional regulation, and nutrient allocation. The integration of molecular marker technologies, CRISPR/Cas-based genome editing, and traditional breeding methods provides a powerful strategy for improving seed size and yield. However, to fully realize the genetic potential of *Brassica napus* in high-yield and high-oil-content breeding projects, further functional validation of candidate genes and a deeper understanding of gene-environment interactions are still needed. With advancements in gene editing technology, combining molecular breeding with traditional interline hybridization is a major trend in modern breeding. This not only improves the genetic diversity of breeding populations but also allows for targeted improvement of related traits, thereby cultivating more practical and groundbreaking *Brassica napus* varieties. Overexpression of *NtHSP17.6* significantly improved the heat and drought tolerance of *Brassica napus*. The transgenic plants maintained better physiological characteristics and growth under abiotic stress conditions, indicating that *NtHSP17.6* has the potential to breed stress-tolerant rapeseed varieties [322]. As shown in Figure 11, the overall structural schematic of this study is presented. Global climate change may lead to more frequent droughts, thereby reducing rapeseed yields. Therefore, rapeseed yields are becoming a challenge, prompting researchers to pay more attention to the crop’s tolerance to harsh environments. To address the future drought stress challenges facing *Brassica napus* L., it is essential to develop drought-resistant varieties and improve cultivation techniques to sustainably increase rapeseed yields. Therefore, the research findings need to be validated in farmland under various climatic conditions; furthermore, management practices also play a crucial role in responding to drought stress. Researchers around the world are exploring various fields, including new variety development, mechanized cultivation, high-oil and high-quality rapeseed processing, green energy, feed powder, and industrial applications. Despite rapid progress in rapeseed application research over the past decade, scientists have yet to determine the quantity and quality of research in this area.

Future research should focus on the following three aspects: (i) cloning genes with high breeding and commercial value and applying for patents; (ii) developing high-throughput platforms for phenotypic analysis, genotypic analysis, and trait analysis; and (iii) integrating genome-wide selection and transgenic technologies to achieve efficient rapeseed breeding and improve growers’ profits.

## Figures and Tables

**Table 1:** Genetic Control of Seed Size: Candidate Genes, QTLs, Molecular Mechanisms, and Their Effects on Seed Traits in Rapeseed.

Standardized Gene Name	*B. napus* Locus ID	Subgenome	Number of Homologous Copies	Evidence Type	Tissue/ Developmental Stage	Loss-of-Function Phenotype	Gain-of-Function Phenotype	Effects on Seed Size/Thousand-Seed Weight/Seeds per Silique/Yield	Validation Status	Main References
*BnaA9.ARF18*	BnaA09g41080D	A9	2 (A9/C8)	2	Silique wall, early seed development	Knockout: longer silique, increased TSW	Overexpression: shorter silique, decreased TSW	Negative regulator; reduces seed weight and silique length; no obvious change in seeds per silique	Validated in *B. napus* via fine mapping and association analysis	[33]
*BnaA9.CYP78A9*	BnaA09g42080D	A9	2 (A9/C8)	1	Silique wall, seed coat	Knockout: smaller seed, shortened silique	Overexpression: enlarged seed and elongated silique	Positive regulator; increases seed size and silique length, elevates thousand-seed weight	Map-based cloning and functional validation in *B. napus*	[55]
*BnaC7.ROT3*	BnaC07g32370D	C7	2 (C7/A7)	2	Silique elongation stage	Knockout: shortened silique	Overexpression: very long silique (VSL)	Positive regulator; promotes silique elongation and indirectly improves seed storage capacity	QTL mapping and candidate gene analysis in *B. napus*	[24]
*BnaC9.SMG7b*	BnaC09g45770D	C9	2 (C9/A9)	1	Anther, ovule, meiosis stage	Knockout: fewer seeds per silique	Overexpression: significantly increased seeds per silique	Regulates seeds per silique (SPS); improves grain number per silique, minor effect on seed weight	Map-based cloning and functional characterization in *B. napus*	[95]
*qSW-A03 (QTL, not gene)*	–	A03	–	2	Seed filling stage	–	–	Major QTL for thousand-seed weight, increases TSW; fine-mapped into a 59 kb interval	Fine mapping; causal gene not yet cloned	[96]
*CqSW.A03-2 (Meta-QTL)*	BnaA03g37960D	A03	–	2	Seed development	–	–	Consensus stable QTL controlling seed weight; candidate gene BnaA03g37960D involved in cytokinin signaling	Meta-QTL integration analysis	[97]
*BnaUBP15*	BnaA06g21020D/BnaC05g23000D/BnaA08g27090D/BnaC08g25720D	A06/C05/A08/C08	4	3	Seed coat, young reproductive organs	Knockout: smaller organ and seed size	Overexpression: enlarged seed	Positive regulator of organ and seed development via ubiquitination pathway	Homology cloning; mainly expression evidence, complete functional verification insufficient	[98]
*BnaDA1*	BnaA02g08820D/BnaC02g12120D	Multiple loci	4	1	Early seed coat development	Loss-of-function mutant: enlarged seed size	Overexpression: restricts seed enlargement	Negative regulator, limits final seed size through protein ubiquitination pathway	Full functional characterization in *B. napus*	[79]
*BnaEOD3*	BnaA01g29810D/BnaC01g32820D	Multiple copies	4	1	Seed coat and silique tissue	Multi-copy CRISPR knockout: larger seeds and longer siliques	Overexpression: increased seed size	Positive regulator promoting cell proliferation; increases seed size and silique length	CRISPR/Cas9 multi-copy mutant validation in *B. napus*	[99]
*BnaARF2*	BnaA03g19400D/BnaC03g22700D	Multiple loci	4	3	Maternal seed coat	Knockout: enlarged seed	Overexpression: smaller seed	Negative regulator for maternal control of seed growth via auxin response pathway	Evidence mainly from expression analysis; lacks complete functional validation	[100]

Note: Evidence Type (uniform across all tables): 1 = Functionally validated in *B. napus*; 2 = Supported by QTL or GWAS trait association evidence in rapeseed; 3 = Supported only by expression data in *B. napus*; 4 = Inferred from orthologous studies in model plants.

**Table 3:** QTL Mapping and GWAS for the Genetic Dissection of Seed Size and Yield-Related Traits in Rapeseed.

Mapping Method	Population Size	Marker Type	Traits Investigated	Major Outcomes	Evidence Type	Advantages	Limitations	Main References
Traditional QTL Mapping	Biparental populations	SSR, SNP, DArT	TSW, silique length	168 QTLs identified	2	High reliability	Low mapping resolution; confined to variation between the two parents	[33]
GWAS	Natural accessions	SNP arrays	Seed weight	Multiple associated loci discovered	2	Captures broad natural diversity; high marker density	Population structure may cause false positives; difficult to detect small-effect loci	[165]
ML-GWAS	403 accessions	High-quality SNPs	8 yield traits	75 stable QTNs detected	2	Improved statistical accuracy; reduces false positives	High computational complexity; sensitive to model assumptions	[166]
QTL-seq	Bulk segregants	NGS-based SNPs	SDPS, VSL	Rapid QTL identification	2	Fast & cost-effective; simple population construction	Poor power for detecting minor-effect QTLs; only works for large phenotypic differences	[167]
Meta-QTL Analysis	Integrated datasets from multiple studies	Consensus markers	Seed weight	6 consensus QTLs identified	2	Cross-study validation; narrows QTL confidence intervals	Quality strongly dependent on original genetic map and data consistency	[32]
BSA-seq	Contrasting phenotypic pools	Whole-genome resequencing	Seed size	204 candidate genes detected	2	Efficient candidate gene screening; rapid localization	Requires strong phenotypic contrast between pools; hard to dissect polygenic minor loci	[168]
Transcriptome-assisted Mapping	Derived from RNA-seq datasets	DEGs	Seed development	Hundreds of DEGs identified	3	Facilitates functional interpretation of candidate regions	Gene expression is highly environment-dependent; association does not equal causal gene	[168]

Note: Evidence Type (same as Table 1).

**Table 5:** Environmental and Physiological Factors Influencing Seed Development, Seed Size, and Yield in Rapeseed (*Brassica napus*).

Environmental Factor	Influence on Trait	Physiological Impact	Associated Genes/Pathways	Evidence Type	Result on Yield	Main References
High Temperature	Reduced oil accumulation in seed	Accelerated seed maturation, shortened grain-filling duration	ABA signaling pathway	3	Lower seed oil content; seed weight may decline	[291]
Drought Stress	Reduced flowering efficiency and seed filling	Restricted assimilate transport from source to sink	Stress-responsive QTLs	2	Significant yield reduction	[292]
Nitrogen Availability	Modulates harvest index	Altered biomass allocation between vegetative and reproductive organs	Nitrogen-use efficiency genes	3	Improved productivity under suitable N supply	[293]
Light Intensity	Alters fatty acid biosynthesis	Enhanced photosynthesis in leaves and silique wall	Lipid metabolism genes	3	Improved oil production under sufficient light	[294]
Rainfall Variability	Disturbs seed-filling process	Changes source–sink balance	QTL-by-environment interaction loci	2	Yield instability across growing seasons	[295]
Soil pH (5.5–8.5)	Influences overall vegetative and reproductive growth	Optimized nutrient uptake capacity	Root developmental genes	3	Optimal plant development within this pH range	[296]
Plant Density	Modifies total pod number per plant	Intra-plant competition for light, water, and nutrients	Yield-related QTLs	2	Direct negative/positive impact on final seed yield	[297]

Note: Evidence Type (same as Table 1).

## Data Availability

The original contributions presented in this study are included in the article. Further inquiries can be directed to the corresponding author.
